# Testing Cancer Immunotherapy in a Human Immune System Mouse Model: Correlating Treatment Responses to Human Chimerism, Therapeutic Variables and Immune Cell Phenotypes

**DOI:** 10.3389/fimmu.2021.607282

**Published:** 2021-03-29

**Authors:** Juan A. Marín-Jiménez, Anna Capasso, Matthew S. Lewis, Stacey M. Bagby, Sarah J. Hartman, Jeremy Shulman, Natalie M. Navarro, Hui Yu, Chris J. Rivard, Xiaoguang Wang, Jessica C. Barkow, Degui Geng, Adwitiya Kar, Ashley Yingst, Dejene M. Tufa, James T. Dolan, Patrick J. Blatchford, Brian M. Freed, Raul M. Torres, Eduardo Davila, Jill E. Slansky, Roberta Pelanda, S. Gail Eckhardt, Wells A. Messersmith, Jennifer R. Diamond, Christopher H. Lieu, Michael R. Verneris, Jing H. Wang, Katja Kiseljak-Vassiliades, Todd M. Pitts, Julie Lang

**Affiliations:** ^1^ Department of Medical Oncology, Catalan Institute of Oncology (ICO-L’Hospitalet), Barcelona, Spain; ^2^ Department of Oncology, Livestrong Cancer Institutes, Dell Medical School, University of Texas at Austin, Austin, TX, United States; ^3^ Department of Immunology and Microbiology, School of Medicine, University of Colorado, Aurora, CO, United States; ^4^ Division of Medical Oncology, School of Medicine, University of Colorado, Aurora, CO, United States; ^5^ University of Colorado Cancer Center, Aurora, CO, United States; ^6^ Division of Endocrinology, School of Medicine, University of Colorado, Aurora, CO, United States; ^7^ Department of Pediatrics, School of Medicine, University of Colorado, Aurora, CO, United States; ^8^ Rocky Vista College of Osteopathic Medicine – OMS3, Rocky Vista University, Parker, CO, United States; ^9^ Department of Biostatistics and Informatics, Colorado School of Public Health, University of Colorado Denver, Aurora, CO, United States; ^10^ Division of Allergy and Clinical Immunology, School of Medicine, University of Colorado, Aurora, CO, United States

**Keywords:** humanized mice, immunotherapy, checkpoint blockade, preclinical model, combination testing, immune correlates, PDX (patient derived xenograft), TME (tumor microenvironment)

## Abstract

Over the past decade, immunotherapies have revolutionized the treatment of cancer. Although the success of immunotherapy is remarkable, it is still limited to a subset of patients. More than 1500 clinical trials are currently ongoing with a goal of improving the efficacy of immunotherapy through co-administration of other agents. Preclinical, small-animal models are strongly desired to increase the pace of scientific discovery, while reducing the cost of combination drug testing in humans. Human immune system (HIS) mice are highly immune-deficient mouse recipients rtpeconstituted with human hematopoietic stem cells. These HIS-mice are capable of growing human tumor cell lines and patient-derived tumor xenografts. This model allows rapid testing of multiple, immune-related therapeutics for tumors originating from unique clinical samples. Using a cord blood-derived HIS-BALB/c-Rag2^null^Il2rγ^null^SIRPα^NOD^ (BRGS) mouse model, we summarize our experiments testing immune checkpoint blockade combinations in these mice bearing a variety of human tumors, including breast, colorectal, pancreatic, lung, adrenocortical, melanoma and hematological malignancies. We present in-depth characterization of the kinetics and subsets of the HIS in lymph and non-lymph organs and relate these to protocol development and immune-related treatment responses. Furthermore, we compare the phenotype of the HIS in lymph tissues and tumors. We show that the immunotype and amount of tumor infiltrating leukocytes are widely-variable and that this phenotype is tumor-dependent in the HIS-BRGS model. We further present flow cytometric analyses of immune cell subsets, activation state, cytokine production and inhibitory receptor expression in peripheral lymph organs and tumors. We show that responding tumors bear human infiltrating T cells with a more inflammatory signature compared to non-responding tumors, similar to reports of “responding” patients in human immunotherapy clinical trials. Collectively these data support the use of HIS mice as a preclinical model to test combination immunotherapies for human cancers, if careful attention is taken to both protocol details and data analysis.

## Introduction

Treatments that block CTLA-4 and/or PD-1/PD-L1 immune checkpoint molecules can release strong anti-tumoral immune responses and have shown important clinical benefit for many malignancies (1–3). Beyond ICB monotherapies, combination immunotherapies, in which typically a targeted drug, chemotherapy or irradiation are co-administered to augment the immune response, have shown strong rationale and are being evaluated in pre-clinical and clinical studies (4–11). However, few are yet standard of care for cancer treatment, indicating there is an urgent need of improving preclinical testing using models that recapitulate the human tumor microenvironment (TME) heterogeneity and anti-tumor immune responses. The TME is a highly complex mixture of tumor, stromal cells and immune cells and their interconnectivity is facilitated by blood vessels, extracellular matrix and signaling molecules (12, 13). The TME is best recapitulated in an *in vivo* setting so testing combination immunotherapies requires animal models. Mouse models have provided the basic tenets for ICB treatments (14, 15). However, syngeneic mouse tumor models represent, at most, a handful of human tumors, and combination studies in these models translate poorly to the clinic (16–19).

Furthermore, the immune systems, drugs and TMEs differ among humans and mice (20–22). Human cancers are heterogeneous, both among and within patients. This cancer diversity is well-represented in clinical trials. However, these trials are very expensive, time-consuming, and wrought with insufficient patient recruitment and high patient variability due to previous treatments and health conditions. In addition, ethical considerations limit tissue accessibility to gather mechanistic data.

For these reasons, scientists have become interested in using Human Immune System (HIS) mice, often referred to as “humanized” mice, as preclinical *in vivo* models to investigate current and to develop novel combinatorial immunotherapies (18, 23–32). HIS-mice are created either by injection of human peripheral blood mononuclear cells (PBMCs), or human hematopoietic stem cells (HSC) into immunodeficient mouse hosts that lack mouse T, B and natural killer (NK) cells due to genetic deletions, including a *recombinase activating gene* (*Rag)* or *Severe-Combined Immunodeficiency (SCID)* mutation and *IL2 Receptor Common gamma Chain* (*IL2RγC)* (33–37). The injection of human PBMCs offers the ability to genetically match the HIS with the tumor (38). However, the developed HIS consists of mostly activated T cells that ultimately mount a human anti-mouse immune response in a classic graft-versus-host reaction (39, 40). Selection of tumor-specific T cells can mitigate this effect; however, the HIS in this case is almost pure T cell lineage cells and omits other important immune cells (38). On the other hand, HIS-mice generated with the engraftment of human HSCs isolated from umbilical cord blood (CB) or fetal liver donor tissue, develop a robust multi-lineage HIS (41–43). Importantly, human tumors grow in these immunodeficient mouse hosts, even in the presence of a HIS developed from an allogeneic CB donor that is non-HLA matched to the implanted tumor (44–46). Furthermore, the HIS that develops in these mice is tolerant of the mouse host. Therefore these “basic”, i.e. that is no thymic transplants or HLA/cytokine transgenes, HIS-models can be used to test human immune responses to a multitude of human cancers that grow in this small animal model. However, the T cell response is not limited to tumor-specific antigens (Ags) as it is also allogeneic.

Since 2016, there have been several reports using the HIS-mouse model to test human immunotherapies (23, 26, 27, 47–50). These studies have provided essential evidence supporting the use of this model to test clinically-relevant treatments on human tumors. However, critical model-specific information is often lacking. HIS-mice suffer from variability in human chimerism that differs over time and among labs (51). Therefore, it is important to consider and report this variability in experimental design and analysis (51). Analyses of human immune cells in tumors alone can be misconstrued without consideration of those cells in the lymph organs of the same animal. In addition, the HIS-mouse model offers the opportunity to perform in-depth characterizations of the immune system. With the abundance of lymph and tumor tissue, one can relatively easily interrogate the activation and functional states of human immune cells, and are not limited to mere subset analyses.

Using a CB-derived HIS-BRGS mouse model (52), we summarize our experiments testing immunotherapy combinations for a variety of human tumors, including breast, colorectal, pancreatic, lung, adrenocortical, melanoma and hematological malignancies. In these experiments, we allocate HIS-mice, implanted with the same patient- or cell line-derived xenografts (PDX or CDX), into multiple treatment arms and study tumor growth and immune responses. Here, we present data from more than 30 experiments to highlight the utility of using the HIS-CB-mouse model for studying ICB-induced changes in immune responses to human tumors. We evaluate the kinetics and subsets of the HIS in lymph and non-lymph organs and consider these data in our experimental protocol for testing immunotherapies. We compare the HIS in lymph organs, which has mouse-to-mouse and time after engraftment variability, to the human immune infiltration in tumors. We show that properties and quantities of tumor infiltrating leukocytes are tumor dependent in this HIS-CB-BRGS model. We further present our flow cytometric analyses of immune cell subsets, activation state, cytokine production and inhibitory receptor expression in peripheral lymph organs and tumors as immune correlates for treatments. We show experiments in which a larger number of human infiltrating T cells are present in responding tumors and that these T cells have a stronger inflammatory phenotype compared to T cells in non-responding tumors, similar to reports of inflammatory signatures in studies of responding patients in human immunotherapy clinical trials (53–55). Collectively these data support the use of HIS mice as a preclinical model to test combination ICB treatments to human cancers, if careful attention is taken to both protocol details and data analysis. Finally, we discuss other applications, including the ability to test tumor-specific responses in HIS-mice, as well as important limitations of these models.

## Materials and Methods

Previously published materials and methods are included in **Supplementary File 1** that details the following sections: CD34^+^ Stem Cell Isolation, Generation of HIS-BRGS Mice, Tissue Harvest and Processing, Cell Staining, Flow Cytometry and Chimerism Evaluation, and ELISA (23, 39, 48, 56).

### Reagents

All reagents used in these studies are listed in **Table 1**.

**Table 1:** Flow cytometry antibodies and reagents.

Antibodies														
Specie	Target		FC	Clone	Vendor	Specie		Target		FC		Clone		Vendor
Anti-human	CD4		BUV395	RPA-T4	BD Bioscience	Anti-human		CD11c		PacB		s-hcl-3		biolegend
	CD45		BUV395	HI30	BD Bioscience			CD45		PacB		HI30		biolegend
	Granzyme B		Fitc	QA16A02	biolegend			CD11c		BV421		s-hcl-3		biolegend
	CD3		Fitc	HIT3a	biolegend			CD25		BV421		M-A251		biolegend
	CD4		Fitc	OKt4	biolegend			PDL1		BV421		29E.2A3		biolegend
	CD5		Fitc	UCHT2	biolegend			Tim3		BV421		F38-2E2		biolegend
	CD8		Fitc	RPA-T8	biolegend			HLA-DR		BV480		G48-6		BD Bioscience
	CD14		Fitc	63D3	biolegend			CD56		BV650		3G8		biolegend
	CD25		Fitc	M-A251	biolegend			CD45RA		BV650		HI100		biolegend
	CD33		Fitc	HIM3-4	biolegend			CD4		BV785		RPA-T4		biolegend
	CD148		Fitc	A3	biolegend			CD19		APC		HIB19		biolegend
	Epcam		Fitc	9C4	biolegend			FoxP3		APC		236A/E7		biolegend
	CD3		PE	HIT3a	biolegend			FoxP3		AF647		259D		biolegend
	CD8		PE	Hit8a	biolegend			Granzyme B		AF647		GB11		biolegend
	CD11b		PE	ICRF44	biolegend			PD1		APC		EH12.2H7		biolegend
	CD14		PE	63D3	biolegend			CD45RA		APC		HI100		biolegend
	CD33		PE	P67.6	biolegend			CD197/CCR7		APC		GO43H7		biolegend
	CD197/CCR7		PE	GO43H7	biolegend			TNFa		APC		Mab11		biolegend
	CD11c		PE	s-hcl-3	biolegend			HLA-DR		APC		L243		biolegend
	CD45RO		PE	UCHL1	biolegend			HLA-DR		APCFire		L243		biolegend
	TIM-3		PE	F38-2E2	biolegend			CD8		APCFire		RPA-T8		biolegend
	Eomes		PE	WD1928	Invitrogen	Anti-mouse		mCD45		APCCY7		30-F11		biolegend
	CD3		PERCP	HIT3a	biolegend			GR1 (Ly6)		PerCP		RBC-8C5		biolegend
	CD4		PERCP	Okt4	biolegend			H-2 (Class I)		PE		M1/42		biolegend
	CD20		PERCP	2H7	biolegend			I-A/I-E (class II)		Fitc		M5/ 114.15.2		
	CD45		PERCP	HI30	biolegend			MHC I-A/I-E		APCCy7		M5/ 114.15.2		biolegend
	CD197/CCR7		PERCP	GO43H7	biolegend			F4/80		AF647		BM8		biolegend
	CXCR3		PERCP	G025H7	biolegend			Ly6G		BV605		IA8		biolegend
	HLA-ABC		PERCP	W6/32	biolegend	Human		FCR Block						miltenyi
	CD3		PeCy7	HIT3a	biolegend	Mouse		CD32				2.4G2		biolegend
	CD11b		PeCy7	ICRF44	biolegend			Zombie Green						biolegend
	CD45		PeCy7	HI30	biolegend			780 Ghost Dye						Tonbo
	IFNg		PeCy7	4S.B3	biolegend									
**Materials**					**Elisa reagents**									
**Name**		**Vendor**			**Type**		**Specie**		**FC**		**Clone**		**Vendor**	
Rec Hu IL6		R&D systems			IgM standard								Sigma	
Rec Hu SCF		R&D systems			IgG standard								Sigma	
Rec Hu FLT3L		R&D systems			Mouse anti-human		IgM		AP		UHB		Southern Biotech	
FCS		Gibco			Mouse anti-human		IgG		AP		JDC-10		Southern Biotech	
FCS		Stemcell			Mouse anti-human		IgM		Unlabeled		SA-DA4		Southern Biotech	
HBSS		Gibco			Mouse anti-human		IgG		Unlabeled		H2		Southern Biotech	
IMDM		Gibco			p-nitrophenly substrate								Sigma	
Golgi Stop		BD Bioscience												
Cell Stim Cocktail		Invitrogen												
Saponin		Sigma												
Formaldehyde		Fisher												
Ficoll Hypaque		GE Healthcare												
DNAase		Sigma												
Liberase DL		Roche												
Pen-strep 100X		Gibco												
Glutamax		Gibco												

FC, fluorochrome.

### PDX and CDX

For ACC, CRC, PDAC and TNBC PDXS: patients undergoing either removal of primary or metastatic cancers at the University of Colorado Hospital were consented in accordance with IRB-approved institutional protocols (IRB #s 08-439, 04-0066, 15-0516). MSI status was verified by PCR in a CLIA-CAP certified lab, where applicable. The PDX models developed at the University of Colorado Denver AMC campus were generated and passaged in athymic nude mice (purchased from Envigo, Hsd : Athymic Nude Foxn1^nu^ Indianapolis, IN) prior to subcutaneous trocar injection into both flanks of HIS-BRGS mice. The SCLC PDXs were obtained through collaborations with Jeffrey A. Kern (National Jewish Health), who obtained original PDX from Dr. Rudin (MSKCC).

TNBC cell line MDA-MB-231 was kindly provided by Scott Kopeck. Melanoma (C8161) and SCLC (H187 and H82) cell lines were obtained from American Type Culture Collection (Manassas, VA). C8161 was modified to express GFP (C8G) and served as a control for C8M line. C8M was engineered to overexpress IRAK-M. DLBCL cell lines OCI-Ly7 and DHL-16 were obtained from Dr. Wing C. (John) Chan (City of Hope Medical Center, Duarte, CA). Thawed aliquots were expanded in DMEM or RPMI media supplemented with 10% FBS, 1% PenStrep and 1% non-essential amino acids. Cell lines were harvested during exponential growth phase within 6 passages, mixed 1:1 with Matrigel (BD Biosciences), and 1-5 million cells were injected subcutaneously into both flanks using a 23-gauge needle. Trocar injection procedure and other development and maintenance instructions for the PDX model are available from https://www.jove.com/v/54393/development-maintenance-preclinical-patient-derived-tumor-xenograft. The cell lines were authenticated by PCR and underwent mycoplasma testing at the Molecular Biology Service Center (Barbara Davis Center, University of Colorado Denver Anschutz Medical Campus).

Experimental details with kinetics and replicates are included in **Table 2**. ICB and combination therapies used for each experiment are included in **Table 3**.

**Table 2:** Experiment nomenclature and details for HIS-BRGS mouse study.

Tumor type	Name	Date(Qtr/Yr)	Tumor take rate(%)	Age at tumor injection(weeks)	Tx start(days)	Age of harvest(weeks)	Exp. length(weeks)	Treatment groups				CB(#)
								Vehicle(n)	ICB(n)	Drug(n)	Combo(n)	
TNBC	A1	03/16	100	17	16	24-25	6-7	3	3	–	–	157
	A2	04/16	88	20-21	10	27	5-6	4	3	2	2	164
	A3	02/17	96	20-23	14-15	23-28	4-6	8	5	–	–	174
	A4	03/17	100	19	19	24	6	4	2	4	3	177
	A5	01/19	93	21-24	21	26-30	5-6	2	2	2	2	194
	A6	04/19	96	16-19	19	21-25	5-6	6	5	–	5	204
	B	01/18	95	21	35	29-32	8-11	3	5	2	4	185
CRC (MSI-H)	A	03/16	89	20	30	25	7-8	6	8	–	–	163
CRC (MSS)	B1	01/17	94	18-21	12	21-24	3	4	4	–	–	171
	C	02/17	100	18	18	22-24	4-6	3	3	2	3	172
	B2	4/2017	94	21-23	17	25-28	4-7	6	4	4	5	182
	D1(P)	03-04/2018	94	18-19	19-41	24-28	5-10	4	5	4	4	187
	E	04/2018	67	19-21	42-61	29-36	9-11	2	5	5	4	192
	F	01/2019	100	21	21	24-26	4-5	6	4	4	6	198
	D2(M)	2/2019	92	18-20	17	22-26	4-5	4	1	3	2	199
	G	3/2019	98	18-22	41-54	27-33	9-11	3	3	4	4	201
	D3(P)	01/2020	98	18-20	20	23-25	5-6	6	–	–	7	206
	H	01/2020	90	19-20	16	23-25	4-6	5	6	6	6	209
	I	02/2020	100	17-20	30	25-30	8-10	6	5	5	6	209
PDAC	A	04/2018	50	31-33	38	37-39	7	1	–	2	–	192
	B	04/2019	90	16-25	25	20-29	5-8	2	–	7	–	206
SCLC	A	01/2019	67	17-24	NA	22-31	5-7	2	–	–	–	198
	B	01/2019	88	18	NA	21-23	3-5	2	–	–	–	198
	C	01/2019	88	15-22	NA	20-25	3-6	4	–	–	–	198
	D	03/2019	100	21-22	19	26-27	5	1	2	2	2	202
ACC	A	03/2017	70	18-19	49-70	29-37	10-18	5	4	–	–	175
	B	02/2020	98	17-21	28-56	26-33	8-12	6	5	6	5	210/212
MEL	A	04/2015	56	15	8	20	6	3	3	–	–	151/152
	B	04/2018	83	21-22	NA	25	3	3	–	–	–	195
	C	04/2018	100	21-22	NA	25	3	3	–	–	–	195
DLBCL	A1	02/2018	100	12	NA	16	4	3	–	–	–	189
	A2	03/2018	100	21	12	25	4	3	3	–	–	194
	B	04/2018	75	26-28	56	30-35	7	3	3	3	3	194

Each row represents an independent experiment with implantation of: triple-negative breast cancer (TNBC), microsatellite stable (MSS) and instable (MSI-H) colorectal cancer (CRC), pancreatic adenocarcinoma (PDAC), small-cell lung cancer (SCLC), adrenocortical carcinoma (ACC), melanoma (MEL) and diffuse large B-cell lymphoma (DLBCL). For each tumor type, we represent different CDX/PDX by a letter, with experimental repeats indicated by a number. (P) refers to a primary tumor and (M) to metastatic origin.

**Table 3:** Human tumor xenografts and treatments in the HIS-BRGS experiments.

Tumor type	Name	CDX/PDX	Cell linename	ICB Drug	CombinationDrug	Tumor Response
TNBC	A1	CDX	MDA-MB-231	Nivolumab	–	Yes
	A2		MDA-MB-231	Nivolumab	HDACi	Yes
	A3		MDA-MB-231	Nivolumab	–	Yes
	A4		MDA-MB-231	Nivolumab	WNTi	Yes
	A5		MDA-MB-231	Nivolumab	WNTi	No
	A6		MDA-MB-231	Nivolumab ± Ipilimumab	–	Yes
	B	PDX	–	Nivolumab	WNTi	No
CRC (MSI-H)	A	PDX	–	Nivolumab	–	Yes
CRC (MSS)	B1	PDX	–	Nivolumab	–	Yes (then relapse)
	C		–	Nivolumab	HDACi	No
	B2		–	Nivolumab	Multi-TKI	Yes
	D1(P)		–	Nivolumab	WNTi	Yes
	E		–	Nivolumab	Multi-TKI	Yes
	F		–	Nivolumab	Multi-TKI	No (all slow growers)
	D2(M)		–	Nivolumab	Multi-TKI	No
	G		–	Nivolumab	WNTi	No
	D3(P)		–	Nivolumab	Multi-TKI	Yes
	H		–	Nivolumab	MEKi + VEGFi	No
	I		–	Nivolumab	MEKi + VEGFi	No
PDAC	A	PDX	–	–	Multi-TKI	No
	B		–	–	WNTi	No
SCLC	A	CDX	H187	–	–	–
	B		H82	–	–	–
	C	PDX	–	–	–	–
	D		–	Nivolumab	Chemotherapy	No
ACC	A	PDX	–	Pembrolizumab	–	Yes
	B		–	Pembrolizumab	Chemotherapy	No
MEL	A	PDX	–	Nivolumab	–	No
	B	CDX	C8G	–	–	–
	C		C8M	–	–	–
DLBCL	A1	CDX	OCI-Ly7	Nivolumab	HDACi	–
	A2		OCI-Ly7	Nivolumab	HDACi	No
	B		DHL-16	Nivolumab	HDACi	Yes

cell-line xenograft (CDX), patient-derived xenograft (PDX), histone deacetylase (HDAC), Wnt signaling pathway (WNT), tyrosine kinase inhibitor (TKI), mitogen-activated protein kinase kinase (MEK), vascular endothelial growth factor (VEGF), “i” represents inhibitor **Materials and Methods**.

### Protocol for Testing Immunotherapies in HIS-BRGS Mice

**Figure 1:**
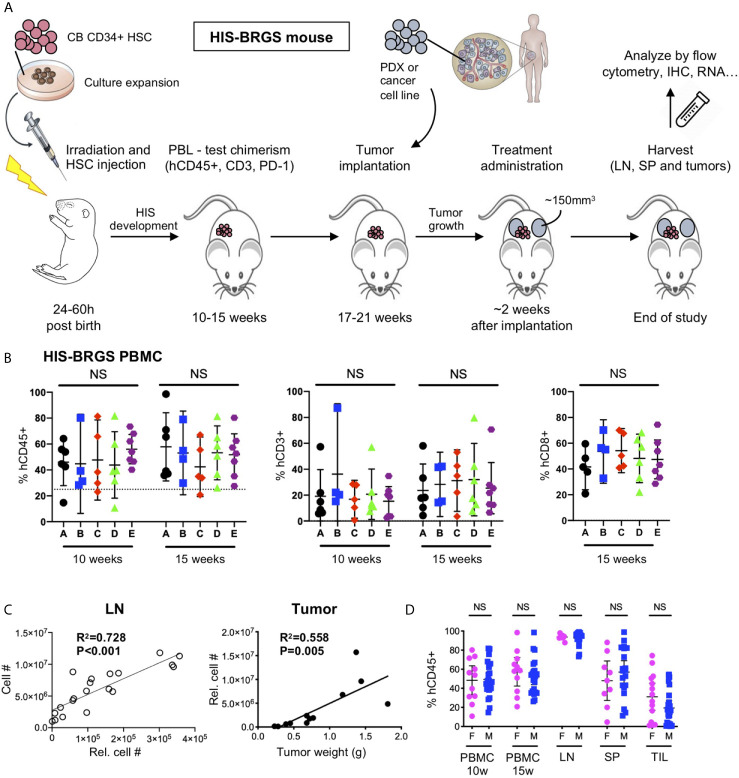
Protocol for evaluation of immunotherapy treatments in HIS-BRGS mice. **(A)** Timeline of HIS-BRGS mouse model development. **(B)** Allocation of HIS-BRGS mice into experimental treatment groups A-E based on equivalent human (hCD45+), T (CD3+) and CD8+ T cell chimerism. NS, no significance [Welch’s ANOVA test]. **(C)** Correlation of cell counts determined by flow cytometry (Relative cell count) and hemocytometer cell count in the lymph nodes (LN, left) or tumor weight (right) [linear regression analysis, R-squared score (R2) and P value (P) in bold if statistically significant (P < 0.05)]. **(D)** Human (hCD45+) chimerism in the blood (PBMC), LN, spleen (SP) and tumor tissue (TIL) of female (F) vs male (M) HIS-BRGS mice [two-group t-test, two tailed].

Model-specific immune attributes have been considered in developing our protocol to study human combination immunotherapies in HIS-BRGS mice. In this protocol, two major characteristics of the model were considered: 1) the engraftment kinetics of the HIS with delayed T cell and LN development, and 2) the inherent variability of the human chimerism. Our experimental timeline is illustrated in **Figure 1A**. At approximately 10 and 15 weeks of age, blood from HIS-BRGS mice *via* retro-orbital route was collected and mixed with 50 μl of heparin. The PBMCs were purified over a Ficoll-hypaque gradient and stained with Abs to mCD45, hCD45, hCD3, hCD19 or hCD20, hCD4 or hCD8, and hPD-1 to assess human chimerism (relative to mouse) as well as T and B cell chimerism, as described previously (23, 57). To reduce the influence of genetic and engraftment variability on experimental outcomes, we allocated HIS-mice (~>25% hCD45^+^) generated from the same CB (see **Table 2** for exceptions) into equivalent treatment groups based on human chimerism, including total T cell and CD8 frequencies (**Figure 1B**). Time of tumor injection was based on human T cell populations, which can vary among cohorts produced from distinct CBs. We also considered the tumor growth kinetics, to coordinate tumor and T cell engraftment with the availability of individual tumors in donor mice. To increase experimental power, we injected tumors into both flanks of the HIS-BRGS mice typically between 19 and 21 weeks of age (**Table 2**). Mice were monitored for health, weighed, and tumors were measured twice weekly and treatments begun once the tumors reached an average volume of 100-300mm^3^. Mice were euthanized in groups of 4-8 based on tumor sizes, health and timing. We attempted to harvest all mice within a reasonable timeframe to gather the most consistent data (**Table 2**). At harvest, we collected lymph nodes (LNs), spleens and tumors, and digested into single cell suspensions, as described in **Supplementary File 1**. Cell counts for LNs (combined peripheral and mesenteric) and spleens were enumerated using a hemocytometer.

### Tumor Growth Calculations

The following equation was used to estimate tumor volume: (length × width^2^) × 0.52 and recorded in the Study Director software package (Studylog Systems). Tumor growth curves are presented as average tumor volume ± SEM for each treatment group in study. The specific growth rate (SGR) was calculated by the following formula:

$$SGR\;(\%\;of\;volume\;growth/day)=\frac{\ln(V_{2}/V_{1})}{\left(t_{2}-t_{1}\right)}$$

### Tumor and Lymph Organ Immune Evaluation by Flow Cytometry

The LN, spleen and tumor cell suspensions are stained with fluorescently-labeled Abs (**Table 1**) to evaluate some or all of the following: 1) mouse (mCD45^+^) and human (hCD45^+^) immune subsets including human T (CD4^+^ and CD8^+^, either through direct staining or defined as CD3^+^CD4^-^ T cells), B (CD19^+^ or CD20^+^), and myeloid (CD11b^+^, CD11c^+^, CD14^+^, or CD33^+^) cells; 2) T cell functional properties including populations of activated T cells (HLA-DR^+^), T_EM_ (CCR7^-^CD45RO^+^) and T_CM_ (CCR7^+^CD45RO^+^) cells, Tregs (CD25^+^, FoxP3^+^), and cytotoxic (Granzyme B (GrB), IFNγ, or TNFα) T cells; 3) CD11c^+^ B cells, 4) the expression of HLA-DR on myeloid cell populations, and 5) the immunogenic properties of tumor (EpCAM^+^ where applicable) and human immune cells, including the expression of inhibitory receptor PD-L1 and MHC class I (HLA-ABC) and II (HLA-DR) molecules. The mCD45 population served as a negative control for human specific expression. We “counted” the number of distinct immune populations on the flow analyzer by staining and collecting a pre-defined volume of cells that we then “back-calculated” to quantitate the total relative cell number (**Figure 1C**). Low chimerism mice (lacking LN or with <1 million human T cells in the spleen at end of the study) were not considered for the analysis.

### Statistics

Data were plotted using GraphPad Prism version 8.3.0 for macOS (GraphPad Software). For comparisons between two independent groups with approximately normally distributed variables, we used unpaired, parametric two-group t-tests with Welch’s correction in the case of unequal variances. One way ANOVA was used to compare means of approximately normally distributed variables among three or more groups, or the Welch’s ANOVA for data sets with unequal group variances. For those variables far from normally distributed, we used a non-parametric Wilcoxon for comparing the median between two groups and Kruskal-Wallis test for more than two groups. Simple linear regression analyses were also performed to assess possible associations using Prism software.

## Results

### HIS Characteristics and Kinetics of Reconstitution in Immunodeficient Mice

**Figure 2:**
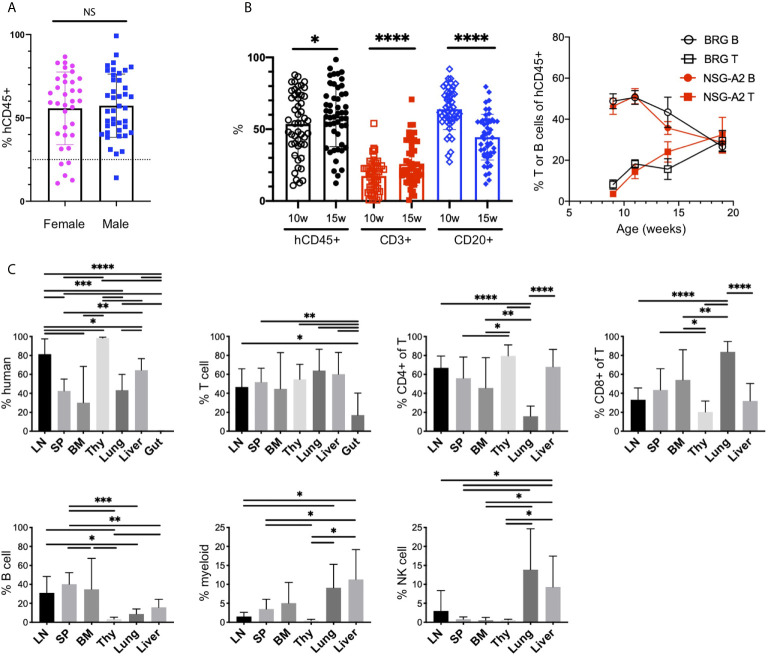
The human immune system in HIS-BRGS-mice. **(A)** Human chimerism (%hCD45 of (h+m) CD45) among female (F) and male (M) HIS-BRGS mice at 10 weeks of age *(n=35 F, n=41 M; two-group t-test p value = 0.74*). **(B)** Percentage of human (hCD45), T cell (CD3) and B cell (CD20) subsets in PBMCs of HIS-BRGS (left panel) and BRG or NSG-A2 mice (right panel) at indicated times post engraftment. *p < 0.05, ****p < 0.0001 [paired t-test, two-tailed]. **(C)** Chimerism by organ for multiple HIS-BRGS mice (n=7, except for BM CD4+/CD8+,n=6) from one CB. T cell (CD3+), B cell (CD19+), myeloid population (CD11b/33/14 or 11c+), NK cell (CD56+) populations. Gate: hCD45+ or hCD45+CD3+ (CD4+, CD8+ of T). *p < 0.05, **p < 0.01, ***p < 0.001, ****p < 0.0001, NS, no significance. [Welch’s two-group t-test, two-tailed between organs as indicated by edges of each line]. *LN, lymph nodes; SP, spleen; BM, bone marrow; Thy, thymus*.

The injection of human CD34^+^ HSCs, isolated from either human CB or fetal liver, into mice lacking T, B and NK cells, and possessing a SIRPα that binds human CD47 (NOD or human), generates robust human chimerism and a multilineage HIS (52, 58, 59). Similar to bone marrow transplantation in humans, the HIS that develops follows a consistent pattern of delayed engraftment and immune reconstitution (56, 60–62). We tested for human chimerism at least 8 weeks post engraftment by collecting blood and enumerating the proportions of mouse (m) and human (h) CD45+ cells by flow cytometry. Human chimerism is variable, with an average percentage of hCD45^+^ > 50% of total hematopoietic (mCD45^+^ + hCD45^+^) cells at 10-15 weeks, consistent with other laboratories (60, 62, 63). In contrast to other reports (62), we observe no difference between chimerism in female and male recipients (55.7 ± 21.8, n=35 females, average 57.2 ± 19.0, n=41 males, p=0.74, **Figures 1D**, **2A**).

B cells are the dominant population early in reconstitution, followed gradually by T cell reconstitution (**Figure 2B**) (51, 56). This pattern is regardless of recipient strain (e.g. NSG, BRG or BRGS), a human HLA selecting element in the thymus (HLA-A2, **Figure 2B**), and even to a large extent the initial age of the recipient mouse (i.e. HSC injection into neonate or adult), although faster T cell reconstitution has been reported from neonatal injections (63). We have previously shown that T cell development is essential for the population of human cells in the LNs, which typically occurs 3-4 months following engraftment and significantly correlates with the presence of human immunoglobulins (Igs) in the sera, a feature consistent with a more functional adaptive immune system (56, 64, 65). We observe relatively high concentrations of hIgG (up to 5 mg/ml) in the sera in our facility (39, 56, 66). Importantly, systemic immune responses are expected in our model, as both T and B cell subpopulations are seen in lymph and non-lymph organs (**Figure 2C** and **Supplementary Figure 1A**).

Cells of the innate immune system, including the myeloid lineage, are important in instructing immune outcomes. HIS-mice in a “basic” NOD/SCID/Il2rγ-null (NSG), NOD/RAG-1/Il2rγ-null (NRG) or BRGS recipient are notoriously deficient in human myeloid cells relative to the lymphocyte lineage (24, 63, 64). However, the mouse myeloid lineage is intact (25, 67, 68). In our HIS-BRGS mice we detected multiple non-lymphocyte human hematopoietic populations by staining with CD11b, CD14, CD33 and CD11c myeloid lineage markers with varying levels of MHC Class II, HLA-DR expression (**Figure 2C** and **Supplementary Figure 1B**). These populations are more prominent in the bone marrow and non-lymph organs (liver and lung) than in the LNs and spleen (**Figure 2C** and **Supplementary Figure 1B**).

We have also examined other less frequent immune populations, and similar to other labs, we have found immature NK cells populations (33, 69–72). We have also observed innate lymphoid cells (ILCs) in low frequencies, mostly in the LNs, that are likely important in LN organogenesis (**Supplementary Figure 2A**) (70). Furthermore, we identified plasmacytoid dendritic cells that we identify as Lin^-^ (CD3^-^CD19^-^CD11b^-^CD11c^-^CD14^-^CD33^-^), HLA-DR^+^ cells with confirmed expression of CD123 (**Supplementary Figure 2B**). We have not observed human mucosal-associated invariant T cells in any organ (73) (**Supplementary Figure 2C**). In our model, we have seen good reconstitution of developing human T cells in the thymus, with both clear double-positive (CD4^+^CD8^+^) and single-positive populations, yet very few human immune cells in the gut (**Figure 2C**) (70).

Overall, the HIS-CB-BRGS model demonstrated strong human chimerism with a multi-lineage immune system, with not only T cells but other essential immune populations, allowing the study of their complex and coordinated interactions during the immune response.

### Human T Cells in HIS-Mice Show an Activated and “Exhausted” Phenotype

Upon Ag encounter, T cells proceed through an activation continuum in which individual cells can ultimately proliferate and produce cytokines (74–76). However, to control this destructive response, immune cells also upregulate inhibitory receptors, leading to a non-responsive state of “exhaustion”. The sequential expression of inhibitory receptors has revealed subsets of exhausted T cells that correlate with the ability to reinvigorate their cytotoxic function: PD-1^+^ Tim3^-^ T cells represent a more “recoverable” population while PD-1^+^TIM-3^hi^ cells are more “exhausted” (74–76). This continuum represents the central paradigm in ICB therapies that are targeting the less-exhausted T cells capable of functioning upon release of the inhibitory signal (75, 76).

**Figure 3:**
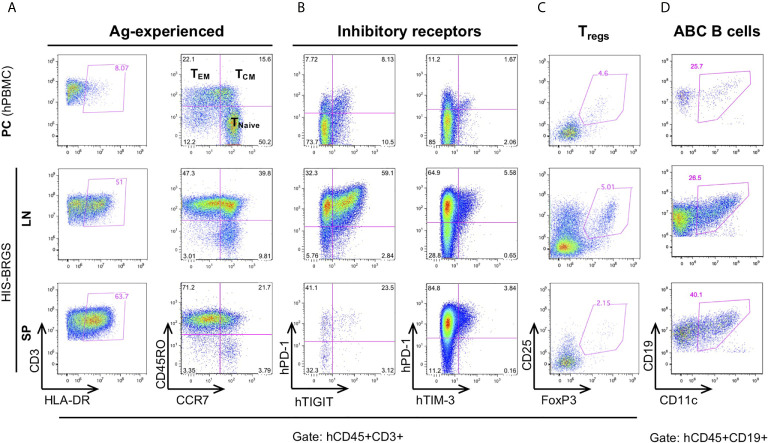
Immunostimulatory and immunomodulating human lymphocyte subsets in the lymph nodes (LN) and spleen (SP) of HIS-BRGS mice. T cell expression of **(A)** activation marker, HLA-DR, and memory cell markers, CD45RO/CCR7, **(B)** inhibitory receptors, PD-1, TIGIT, TIM-3, and **(C)** regulatory T cell markers, CD25^+^FoxP3^+^. **(D)** CD11c^+^ B cells. Human PBMC served as a technical staining control (“PC”, top row).

Relevant to the HIS model, we find extensive T cell activation, as determined by HLA-DR expression, and populations of both effector memory (T_EM_) (77) and central memory (T_CM_) T cell populations (56) (**Figure 3A**, **Supplementary Figure 3A**). In addition, we observe a very large population of T cells with an exhausted phenotype, as defined by expression of both PD-1 and TIGIT inhibitory receptors that increases with time (23, 48, 78). However, these same T cells show weak co-expression of TIM-3 (**Figure 3B**). The expression of Eomes and T-bet transcription factors in T cells from lymph organs and tumor infiltrating leukocytes (TILs) of HIS-BRGS mice also support an activated and exhausted gene expression profile in these mice (**Supplementary Figure 3B**). Furthermore, we consistently observe obvious regulatory T cell (Treg) populations, which are more notable in the LNs than in the spleen (**Figure 3C**). The prevalence of this population in HIS models has been inconsistently reported, perhaps due to experimental timing or differences in anatomical location (51, 79, 80). These activated/exhausted T cell phenotypes correlate with increased frequencies of CD11c^+^ B cells, a population associated with increased Ig production in both humans and mice (**Figure 3D** and **Supplementary Figure 3A**) (81–83).

In view of these results, we can confirm the activated phenotype of T and B cells in our model, as routinely seen in untreated cohorts of both unmanipulated and tumor-bearing HIS-BRGS mice (23, 48, 56).

### Growth of Human Tumors in HIS-Mice

A major breakthrough for the HIS model was the discovery that tumors can grow in humanized mice (29, 46, 47). Indeed, our group has validated this finding using flank injection models of multiple human cancers (23, 48, 49). **Table 2** provides experimental details for each of our 33 HIS-BRGS oncology studies, including nomenclature, study time points and number of mice per treatment group. **Table 3** summarizes the origin of the 33 tumors (PDX/CDX and tumor type), study treatment for each experiment, usually ICB +/- combinational drug, and whether significant tumor growth inhibition was measured.

As an indication of reproducibility of the system, we injected the same MDA-MB-231 TNBC CDX in six independent HIS-BRGS experiments, with six distinct CBs (**Table 2**, A1-A6). In all of these experiments the tumors grew quickly, and similar to rates observed in nude mice or non-humanized BRGS recipients (23). Of note, tumor take rate for all 33 experiments was high, with a mean of 89.4% (95%CI 84.6 – 94.1) and did not significantly differ across tumor types (*P*=0.18) or xenograft class (CDX vs PDX, *P*=0.53). As illustrated by CRC experiments, tumor take rate was also similar between primary tumor and metastatic tissue derived PDXs (**Table 2**, CRC D1-D3).

Although we typically use a flank-injection model for ease of measurement and consistency, the model is well-suited for orthotopic injections, which has been shown to yield results consistent to the flank injection model in a similar HIS-mouse model (44). We have observed metastatic spread to the liver and lungs in some tumors, and also in EBV-induced lymphomas that developed *de novo* following viral infection (66). Indeed, we have recently observed metastatic liver burden from i.v. injection of a CDX, in which we could observe tumor growth using the *in vivo* imaging system (IVIS, data not shown).

In summary, we provide ample evidence that human xenografts grow in the multi-lineage HIS models generated from human HSCs. As similarly observed by other groups (50, 84), we achieved high and reproducible tumor take rates in our HIS-BRGS mice.

### Infiltration of Human Immune Cells in HIS-BRGS Mice Varies by Tumor Type

**Figure 4:**
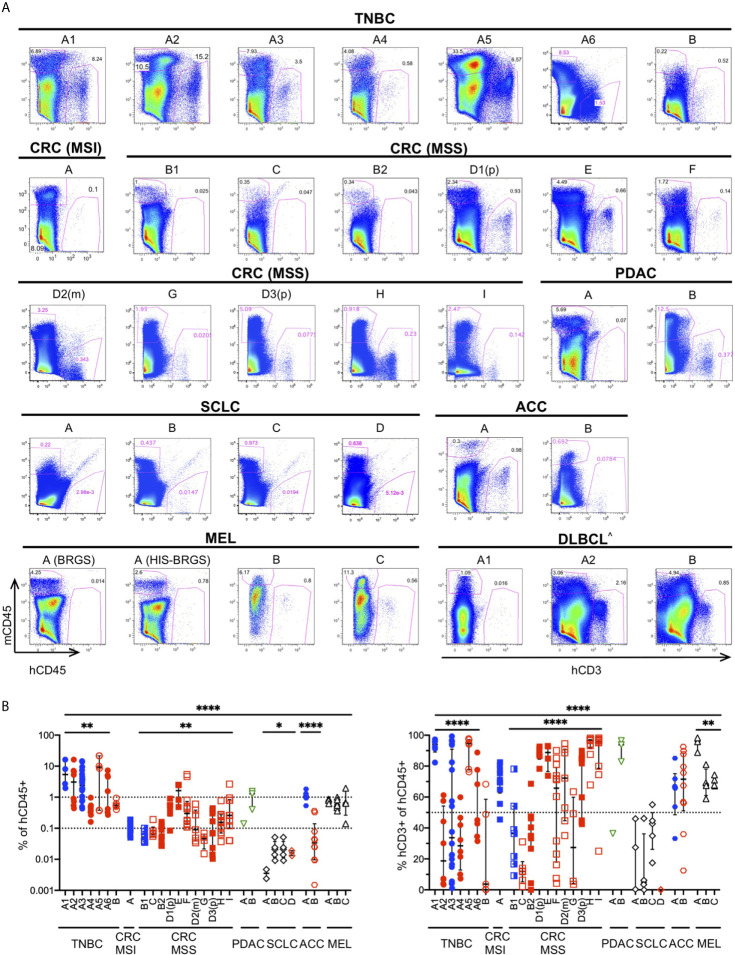
Infiltration of human immune cells into different human tumors in **untreated** HIS-BRGS mice. **(A)** Representative flow cytometry plots showing mouse (mCD45^+^, y-axis) and human (hCD45^+^ or (^) hCD3^+^ for hematological malignancy, x-axis) leukocyte infiltration into tumors in HIS-BRGS mice: TNBC MDA-MB-231 (A1-6) and PDX **(B)**; CRC PDX: MSI-H **(A)** and MSS (B-I); PDAC PDX (A, B); SCLC CDX **(A, B)** and PDX (C, D); ACC PDX (A, B); melanoma PDX **(A)** and CDX (B,C); and DLBCL CDX (A,B). Different experiments with the same tumor have the same letter but different numbers; (p) refers to a primary tumor and (m) to metastatic origin. Background staining represented by melanoma PDX A injected into non-humanized BRGS mouse (lower left). **(B)** Percentage of hCD45^+^ (of singlet gate, left) and CD3^+^ T cells (hCD45+ gate, right) in tumors of untreated HIS-BRGS mice. Filled symbols represent experiments in which tumors in treated HIS-BRGS mice responded to treatment and open symbols represent non-responding experiments. The data are log-transformed to approximate normal distributions. The colors represent experiments testing nothing (black), ICB monotherapy (blue), targeted therapies alone (green) or immunotherapy combinations (red). *p < 0.05, **p < 0.01, ****p < 0.0001 [The lower lines in B reflect analyses of differences in means across experiments of the same tumor type using Welch’s ANOVA (TNBC, CRC, SCLC, MEL) or two-group t-test (ACC). The upper line shows Welch’s ANOVA analysis for differences in means across all tumors, regardless of type].

The TME governs the immune response against malignant cells. “Cold” tumors have reduced immune infiltration and an immune-suppressive TME while “hot” tumors are characterized by cytotoxic T cell infiltration (12, 85). To ascertain if the degree of infiltration was determined by differences in the chimerism among HIS-BRGS mice or was rather influenced by the tumor itself, we measured the infiltration of immune cells into the tumors in these thirty-three independent experiments in which a human CDX/PDX was implanted into the flanks of HIS-BRGS mice (**Table 2**). We found different patterns of immune infiltration, as shown in the variable mCD45^+^ and hCD45^+^ proportion across different experiment examples (**Figure 4A**). In order to achieve a general overview, we then plotted cumulative hCD45^+^ and hCD3^+^ percentages for all vehicle, i.e. untreated, HIS-BRGS mice in each of these experiments (**Figure 4B**). We observed variable infiltration of human immune cells within single experiments (e.g. TNBC A1), but larger differences in average infiltration were observed across tumor types (**Figures 4A, B**). For instance, the infiltration into the TNBC CDXs A1-6 was variable (range 0.16-76.6, p=0.0032 Welch’s ANOVA) but the average infiltration was consistently higher than our low-infiltration tumor “cut-off” of 0.1% cell, which in our experience represents samples with too few human cells to perform further analyses (<100 hCD45+ cells). In comparison, the CRC MSS PDXs had relatively low infiltration of hCD45^+^ cells with similar variability among tumors (range 0.015-5.38, p=0.0028 Welch’s ANOVA) compared to the TNBC data sets. However, the average infiltration for all TNBC CDXs (A1-A6) versus all CRC MSS tumors (B1-I) showed a significant difference (p =0.0015, two-group t-test with Welch’s correction). The conclusion holds, with even more significance, after removing the two highest TNBC outliers (>70%) with p<0.0001 using the same test. The experiments with implanted tumors of shared xenograft origin showed similar hCD45^+^ infiltration, as illustrated by CRC-MSS PDXs “B1/B2” (p=0.31, two-group t-test) and “D1p/D2m/D3p” (p=0.10, Welch’s ANOVA test) although D1p and D3p did show variable infiltration of hCD45+ cells (p=0.04, two-group Welch’s t-test). These differences among PDXs of same origin are small relative to differences measured across all CRC MSS tumor types. The percentage of hCD45^+^ also mirrored the expected infiltration for other tumors. For example, the MSI-H Lynch syndrome-associated ACC tumor “A” showed higher hCD45^+^ infiltration relative to the sporadic ACC “B” counterpart. Indeed, SCLC tumors “A-D” had almost no hCD45^+^ infiltration as expected for this well-known “cold” tumor (86, 87). We also compared right versus left flank tumors and observed quite consistent hCD45^+^ infiltration within an individual HIS-mouse, although the tumors were often quite different in size (**Supplementary Figure 4**) (44, 84).

As a further indicator of TME phenotype, we measured the frequency of human T cells in these same tumors (**Figure 4B**). We observed much greater variability in T cell frequencies, among and within tumors of different origins. Even among the few hCD45^+^ cells detected in the SCLC tumors, there were different amounts of T cell infiltration. On the other hand, melanoma tumors had consistently high T cell infiltrates. Similar to reports by other groups (50), we found very few infiltrating B cells, although this lineage is prevalent in the spleens and LNs of HIS-BRGS mice (23). The majority of the non-T cells in tumors of our HIS-BRGS mice was of the myeloid lineage (23). Altogether, and consistent with other studies in HIS-mice (44, 84), these data suggest the tumors in our HIS-BRGS model generally recapitulate their unique TME in regard to immune infiltration.

### Parameters Influencing Human Infiltration

Human immune cells clearly infiltrated human tumors implanted into HIS-mice. To better understand the factors that could influence tumor infiltration in our model, we analyzed the data with respect to protocol parameters.

#### Influence of Human Chimerism on Tumor Immune Infiltration

**Figure 5:**
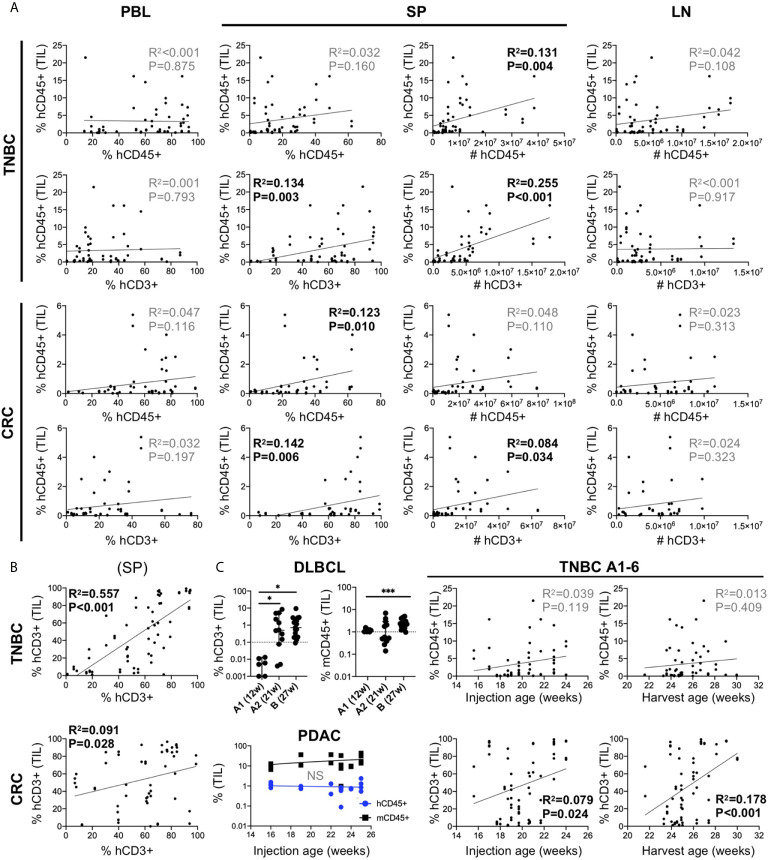
Influence of human chimerism and timing of tumor injection on human immune infiltration in human tumors in untreated HIS-BRGS-mice. **(A)** Correlation of hCD45^+^ infiltration in HIS-BRGS tumors with human chimerism in lymph organs. Data from six TNBC CDX (top, A1-A6) and five CRC (bottom, CRC B2, D2, D3, E, F) independent experiments. hCD45 and hCD3 chimerism (percentage and absolute number, #) in PBMC prior to tumor injection (PBL at 15 weeks), and in the spleen (SP) and lymph nodes (LN) at end of study. **(B)** Positive correlation between T cell infiltration in TNBC A1-A6 and CRC tumors (B2, D2, D3, E, F) and T cell chimerism in spleens of HIS-BRGS mice. **(C)** Influence of timing of tumor injection on human immune cell infiltration into tumors. Human (hCD45+) or T (hCD3^+^) and mouse (mCD45^+^) immune cell infiltration into DLBCL CDXs (*upper left*) or PDAC PDX (lower left) with different tumor injection timepoints. Correlation of hCD45+ and hCD3^+^ infiltration into untreated TNBC CDXs (A1-A6) and the age of HIS-BRGS mice at time of tumor injection or end of study (*right*). *p < 0.05, ***p < 0.001 [**A, B**: Linear regression analysis, R-squared score (R^2^) and P value (P) in bold if statistically significant (P < 0.05); **(C)**: Welch’s two-group t-test, two-tailed between organs as indicated by edges of each line].

Given the variable nature of human chimerism in HIS-mouse models, we sought to determine the influence of this feature on infiltration into the tumors. First, we correlated tumor hCD45+ infiltration with the amount of hCD45^+^ and hCD3^+^ cells measured in 1) PBMCs of the HIS-mice prior to tumor injection (we allocated mice into equivalent “chimerism” groups based on this parameter) and 2) the spleen at end of study. We analyzed combined data from six TNBC CDX (A1-6) and five CRC PDX (B2, D2, D3, E, F) independent experiments (**Figure 5A**). Tumor immune infiltration did not appear significantly influenced by human chimerism or human T cell percentage, as determined in the blood of the HIS-mice prior to tumor injection. However, tumor hCD45^+^ infiltration showed a positive correlation with human chimerism at the end of study, in terms of total number (TNBC) or percentage (CRC) of hCD45^+^ cells in the spleen of HIS-mice. In contrast, the number of human cells in the LNs, which are consistently >85% hCD45+ cells, showed no correlation with hCD45^+^ tumor infiltrates in the either models. Notably, we observed a stronger correlation of hCD45^+^ tumor infiltration with the percentage and number of T cells in the spleen, most notable in TNBC A1-A6 tumors (% T cells p=0.003, T cell #s p<0.001), but also among the CRC MSS PDX models (% T cells p=0.006, T cell #s p=0.034). Similarly, the proportion of T cells in the spleen correlated with those in the tumors with a higher correlation in the TNBC than CRC MSS datasets (**Figure 5B**). In our studies, hCD3^+^ rather than hCD45^+^ percentage in the blood prior to tumor injection appeared as a better indicator of respective chimerism in the spleen (and thus T cell infiltrates in tumor) at end of study (**Supplementary Figure 5**). Overall, we did not detect a clear impact of baseline chimerism on tumor immune infiltration, but our data suggests that the systemic response seen in the spleen, which correlates with T cell chimerism in the blood, may be influencing tumor infiltration.

#### Timing of Tumor Injection

T cells take months to appear in immune organs in HIS-mice, and yet they influence human immune cell infiltration into the tumors. Therefore, we evaluated the influence of timing of tumor injection on human infiltration into tumors. In a series of experiments using a DLBCL CDX, the tumors were injected at 12 (“A1”) or 21 (“A2”) weeks (**Table 2**). The DLBCL is of hematopoietic origin and express hCD45 and hCD19, thus we evaluated the infiltration of human T cells (CD3^+^) as the readout for human immune infiltration. In the case of early tumor injections, we observed very few tumor-infiltrating T cells, although mCD45^+^ (myeloid lineage) cells clearly infiltrated the tumors. However, in mice injected at 21 weeks of age, we observed increased frequencies of hCD3^+^ T cells in the tumors, confirming the need for later tumor injections. DLBCL CDX “B”, that was injected at a much later timepoint (27 weeks), showed similarly high frequencies of CD3^+^ T cells (**Figure 5C**). In another experiment, PDAC tumors were injected into a cohort of HIS-BRGS mice of different ages (16-25 weeks). In this case the human infiltration remained consistently low, likely influenced more by a suppressive TME even in the presence of sufficient human T cells (**Figure 5C**). Consistent with the correlation of increased splenic hCD3^+^ T cells with hCD45^+^ infiltration into tumors in the MDA-MB-231-bearing HIS-mice (see section 5.1), a similar analysis showed increased human T cell infiltration in these tumors with either age of tumor injection and age of mice at end of study (**Figure 5C**). Thus, we conclude that, in addition to a basic human chimerism cutoff, it is also important to: 1) have sufficient T cells in the HIS-mice for the tumor studies (i.e. timing of tumor injections is crucial) and 2) treatment cohorts must be allocated by human and more importantly, T cell PBMC chimerism, to reduce potential effects of chimerism on experimental outcomes.

#### Cord Blood

The generation of HIS-mice from distinct CB units is another source of variability that should be considered. Our TNBC and CRC studies testing the same tumor with different CBs suggest the CB plays a relatively minor role in determining human infiltration (**Figures 4A, B**, TNBC A1-A6, CRC B1-B2, and CRC D1-D3). In addition, we provide two examples below (see section 7) to show differences in immune responses to two unique CRC PDX or DLBCL CDX HIS-BRGS models that were treated with the same therapies in mice generated from the same CB.

### Immune Response to Tumor Allograft in HIS-BRGS Mice

Despite high and reproducible tumor take rates in our HIS-BRGS mice (50, 84), tumor sizes varied greatly (**Supplementary Figure 4**), a feature that is also common in mouse syngeneic studies (88). We wondered if the HIS anti-tumoral response contributes to this variability. Since time points differed between experiments, tumor weight or volumes measured at a specific time do not accurately reflect tumor growth in our study. Therefore, we calculated the specific growth rate (SGR, see material and methods section) for each tumor, which represents the growth of the tumor considering initial and ending times and volumes.

**Figure 6:**
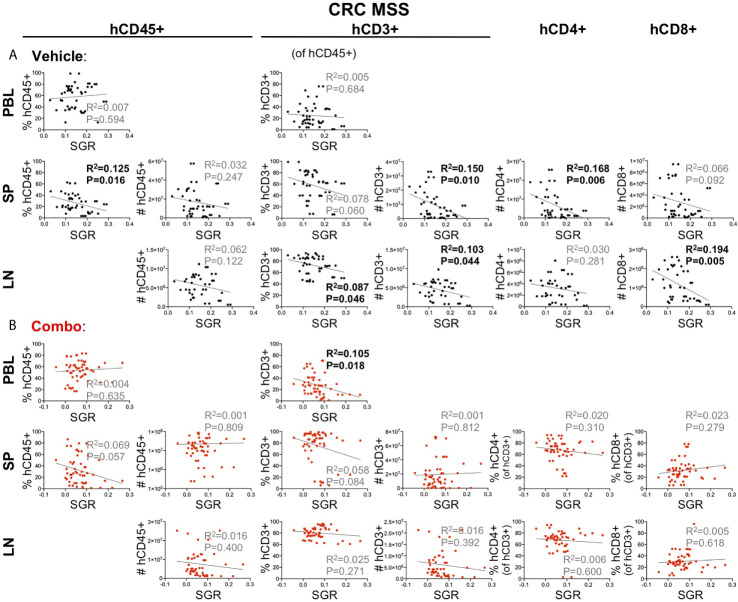
Correlation of tumor growth and human chimerism in lymph organs for 5 CRC MSS PDX models (B2, D2, D3, E, F) in HIS-BRGS mice receiving either no treatment **(A)** or a combination immunotherapy **(B)**. Linear regression analysis was performed between tumor specific growth rate (SGR) and immune cell populations in the blood prior to tumor injection (PBL at 15 weeks) or in the spleen (SP) and lymph nodes (LN) at end of study. [Linear regression analysis, R-squared score (R^2^) and P value (P) in bold if statistically significant (P < 0.05)].

We first analyzed the effects of the HIS on the SGR in individual vehicle (untreated) mice from five CRC experiments (B2, D2, D3, E, F) (**Figure 6A**). We observed no difference in the growth of tumors relative to the hCD45^+^ or T cell chimerism in the blood prior to tumor injection or number of human cells in the LNs or spleen at end of study. However, we did notice a negative correlation between SGR and percentage of hCD45^+^ cells in the spleen and number of T cells in secondary lymph organs. Indeed, the number of CD8+ T cells in the LNs and CD4+ T cells in the spleen were the strongest predictors of slower-growing tumors in our analysis. Thus, we speculate that a T cell-driven response may be influencing tumor growth in HIS-BRGS mice; however, this effect is relatively marginal as the tumors continue to grow, albeit at different rates.

In this same series of CRC experiments, we secondly analyzed HIS-BRGS mice that had been treated with an ICB combination therapy for at least two weeks (**Figure 6B**). In this case, we found that the frequency of T cells in the PBMCs prior to tumor injection correlated with tumor growth rates. In these treated mice, the tumors were overall smaller in size, with lower SGRs, supporting a successful combination ICB therapy response (manuscript in preparation). In this ICB-treated cohort, the human chimerism in the immune organs at end of study had no significant influence on the tumor growth rates. Thus, the effect of T cell responses on tumor growth in untreated HIS-BRGS mice is not that obvious in ICB-treated HIS-BRGS tumor-bearing mice, where an overwhelming immune response might be masking its influence.

### Correlates of Immune Response

**Figure 7:**
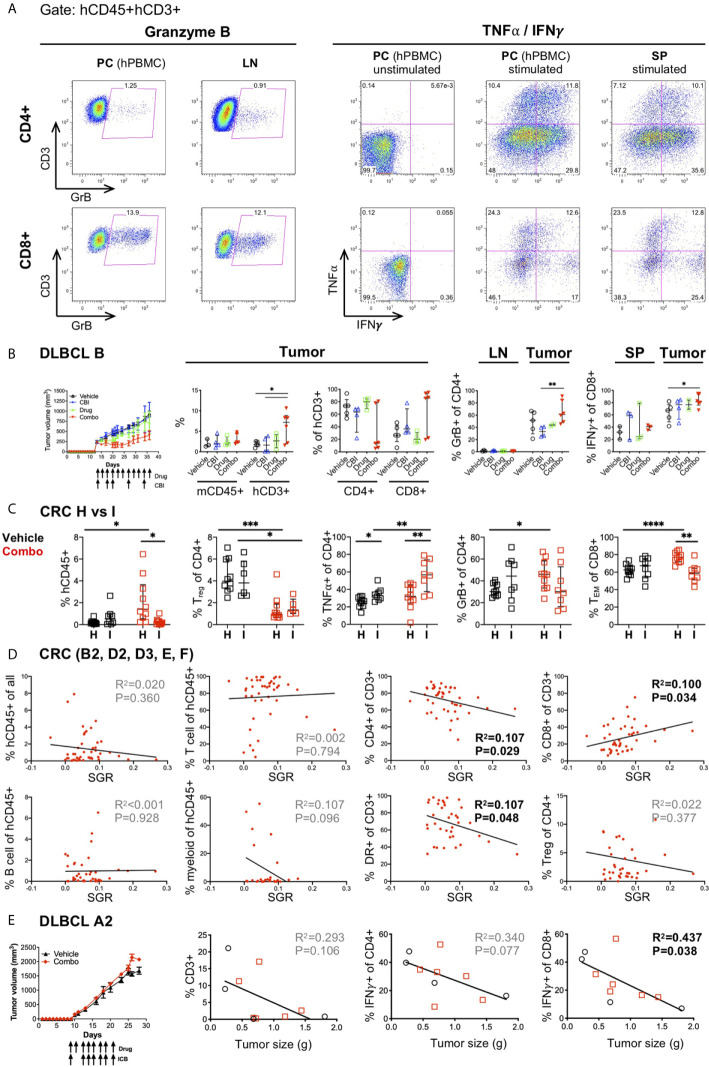
Correlates of immune response to human tumors following combination immunotherapy treatments in HIS-BRGS mice. **(A)** Representative flow cytometry plots showing expression of Granzyme B (left) and TNFα and IFNγ cytokines (right) in CD4^+^ (top row) and CD8^+^ T cells (bottom row). Cells are cultured overnight with cell stimulation and blocked with Golgi Plug for final four hours to detect TNFα and IFNγ. Human PBMCs serve as technical staining control, and as stimulation controls for TNFα/IFNγ. **(B)** Tumor growth and immune response in HIS-BRGS mice with DLBCL CDX B tumors treated with monotherapies and combination ICI therapies. Tumor growth curves over time (left panel) and frequencies of mCD45^+^, hCD3^+^, CD4^+^ T, and CD8^+^T cell populations (left graphs), and GrB^+^CD4^+^ T cells and IFNγ^+^CD8^+^ T cells (right graphs) in indicated tissues. **(C)** Immune correlates of combination immunotherapy response to two CRC MSS PDXs in HIS-BRGS mice, generated from same CB. Infiltration of hCD45^+^ cells (left), and frequencies of Tregs, TNFα^+^CD4^+^ T, GrB^+^CD8^+^ and T_EM_ CD8^+^ cells in CRC (H, I) PDXs in HIS-BRGS mice. **(D)** Correlation of immunotypes and tumor growth (SGR) in a series of five independent experiments of HIS-BRGS mice bearing CRC MSS PDXs (B2, D2, D3, E, F) and treated with the same combination immunotherapy. The frequency of human (hCD45^+^), T (CD3^+^), CD4^+^ T, CD8^+^ T, B (CD19^+^), and myeloid (CD11b^+^, CD11c^+^, CD14^+^ or CD33^+^) cells (top rows), and the frequency of activated (HLA-DR^+^) T cells (2^nd^ row, 3^rd^ panel) and Tregs (2^nd^ row, 4^th^ panel) in CRC PDX relative to the specific growth rate (SGR) for that tumor. **(E)** Tumor growth and immune correlates in HIS-BRGS mice with DLBCL CDX A2 tumors and treated with same combination ICI therapy as in **(B)**. Tumor growth curves over time (left panel) and correlations of tumor size with frequency of CD3, IFNγ CD4^+^ and IFNγ CD8^+^ in DLCBL A in HIS-BRGS mice. Symbols represent data from an individual tissue from tumor-bearing HIS-BRGS mouse that are untreated (black), treated with ICB (blue) alone, drug alone (green), or combination ICB (red). *p < 0.05, **p < 0.01, ***p < 0.001, ****p < 0.0001 reflects significance test between two groups at edge of line. [**(B,C)**: Two-group t-test with Welch’s correction, two-tailed; with the exception of % mCD45 and % of hCD3+ (CD4+ and CD8+) in the tumor, which was evaluated with non-parametric test due to confirmed non-normal distribution in the “combo” group. **(D, E)**: linear regression analysis, R-squared score (R^2^) and P value (P) in bold if statistically significant (P<0.05)].

A major objective in cancer immunotherapy studies is to understand why some patients respond and others do not, in an effort to define predictive biomarkers of response and prognosis. Many recent high-impact studies have focused on determining immune properties that correlate with therapeutic response (53–55, 89–94). These studies have been performed in mouse models and/or clinical trials using PBMCs and rarely tumor tissue following treatments. With our tissue availability, we designed multiple flow cytometry panels, to evaluate 1) the immune subsets in the HIS-mice (**Supplementary Figure 1**), 2) the activation state of the T cells including the upregulation of HLA-DR and PD-1 or TIM-3 inhibitory receptors, as well as the frequency of Treg, naïve, T_EM_ and T_CM_ populations (**Figure 3**), 3) the cytotoxic T cell effector function as determined by production of GrB, TNFα and IFNγ (**Figure 7A**), and 4) Ag presentation molecules and inhibitory receptors on the tumors (see section 8) (23).Using these flow panels, we have analyzed immune correlates in two ways: 1) by treatment groups or 2) by tumor response (SGR).

In previous studies we used the first approach to establish immunotypes among anti-PD-1 treated HIS-BRGS mice (23, 48, 49). Here we apply this strategy to immune responses following combination immunotherapies. We observed increased frequencies of hCD3^+^ T cells in the tumors of DLBCL B CDX from mice in the combination-treated group. Furthermore, we observed trends of increased CD8+ T cells, GrB^+^CD4^+^ and IFNγ^+^CD8^+^ among the T cells infiltrating the combo-treated tumors, although the data were not statistically significant in all cases among this small sample with variable responses among the combination-treated group (**Figure 7B**). As stated earlier, another likely source of variability in our system is the CB. Human HSCs harbor genetic variation and we have found that the human chimerism, both the kinetics and amounts, can differ by CB batch. We have generated >60 mice from some CB HSCs, and in these cases, we have used HSCs from the same CB for different experiments. Two distinct CRC MSS PDXs (“H” and “I”) were implanted in mice generated from the same CB, and then treated with the same drugs. We observed similar human infiltrations (see H and I in **Figures 2A, B**), yet distinct immune responses (**Figure 7C**). Using the first approach, only “H” CRC model showed increased human infiltration upon combination immunotherapy. In contrast, we observed a significant decrease in Treg frequencies in both PDXs after treatment. Remarkably, PDX “I” showed increased TNFα^+^ CD4^+^ T cells whereas PDX “H” had enriched GrB^+^ CD4^+^ and T_EM_ CD8^+^ populations.

Using the second (SGR) approach, we sought to elucidate whether an immunotype, as opposed to the human chimerism (see section 6 and **Figure 3**), was associated with lower tumor growth rates. This approach is more akin to studying immune correlates of responders versus non-responders in clinical trials. We used the series of five CRC PDXs (B2, D2, D3, E, F) treated with the same combination immunotherapy for this analysis. We established that activated HLA-DR^+^ CD4^+^ T cells in the tumors were the best predictors of tumor growth inhibition, in line with the analyses by treatment groups (**Figure 7D** and manuscript in preparation). Interestingly, using this same approach to evaluate an innate response to the tumors by the HIS in untreated mice in this model, revealed that a lower SGR correlated with increased IFNγ^+^ CD4^+^ T cell and decreased myeloid and TNFα^+^ CD8^+^ immunotype (**Supplementary Figure 6**). We further assessed the CB effect by studying two DLBCL CDXs (“A2” and “B”) implanted into HIS-BRGS mice generated from the same CB. We found that CDX “B” had tumor growth inhibition upon combination immunotherapy (**Figure 7B**) while CDX “A2” did not (**Figure 7E**). By analyzing the T cell infiltration and frequency of IFNγ^+^ CD4 and CD8 T cells for model “A2”, we found that increased T cells in the tumors, most notably the IFNγ^+^ CD8 T cells, correlated with slow-growing tumors (**Figure 7E**). In this experiment, one of the three untreated mice appeared to have robust tumor growth inhibition in both flanks, illustrating the need for sufficient number of mice per treatment group.

Thus, we demonstrated that we can observe immunotypes that correlate with drug regimens and tumor responses in our HIS-BRGS tumor-bearing mice. Notably, these immunotypes can be unique to different tumors or combination treatments, even with the same CB.

### Evaluation of Tumor Immunogenicity

**Figure 8:**
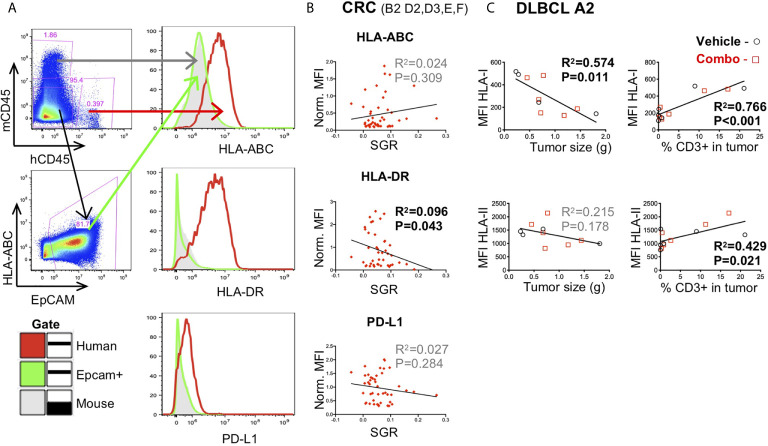
Expression of immune-related molecules on human tumors in HIS-BRGS mice. **(A)** Representative flow cytometry plots illustrating gating for expression on the tumors: mCD45^-^ hCD45^-^ cells are gated in the tumor cell suspension and when possible, a tumor-specific Ag (e.g. EpCAM) is included, **(B)** Correlation of tumor growth rates with expression of human HLA-ABC (MHC Class I), HLA-DR (MHC Class II) and PD-L1 on hCD45^-^mCD45^-^EpCAM^+^ CRC MSS PDX (B2, D2, D3, E, F) in HIS-BRGS mice. **(C)** Correlation of tumor weight (grams) and human T (hCD3) cell infiltration with expression of human HLA-ABC (Class I) and HLA-DR (Class II) on DLBCL CDX A2 in HIS-BRGS mice. Expression of HLA-ABC, HLA-DR and PD-L1 on hCD45^+^ and mCD45^+^ populations serve as positive and negative controls, respectively. [Linear regression analysis, R-squared score (R^2^) and P value (P) in bold if statistically significant (P < 0.05)].

The TME plays an active role in suppressing the immune system, oftentimes by decreased Ag presentation. To evaluate the effect of treatments on the TME in our HIS-BRGS mice, we measured the expression of both class I (HLA-ABC) and II (HLA-DR) MHC molecules on the tumor cells by flow cytometry. We consistently observed low MHC I and II expression on human cells in tumors, and even more so on the tumor cells themselves (**Figure 8A**) (23, 48). However, we have previously reported upregulation of HLA-ABC and HLA-DR on tumors following immunotherapy treatment (23). We observed both increased MHC class II expression in slow-growing CRC MSS models (**Figure 8B**) and increased MHC class I expression in smaller and T cell-enriched DLBCL “A2” tumors (**Figure 8C**). In these DLBCL tumors, increased IFNγ expression by CD8 T cells similarly correlated with slow-growing lymphomas, consistent with reports showing upregulation of MHC genes by IFNγ responsive genes and increased tumor response (**Figure 7E**) (95–97). Furthermore, ICB success has been related to PD-L1 expression in the tumor for some tumor types (98), so we measured the relative MFI expression of PD-L1 inhibitory receptor on the tumor cells and found no significance in the CRC MSS models (**Figures 8A, B**).

### Mouse Immune Myeloid Subsets in Tumors of HIS-BRGS Mice

**Figure 9:**
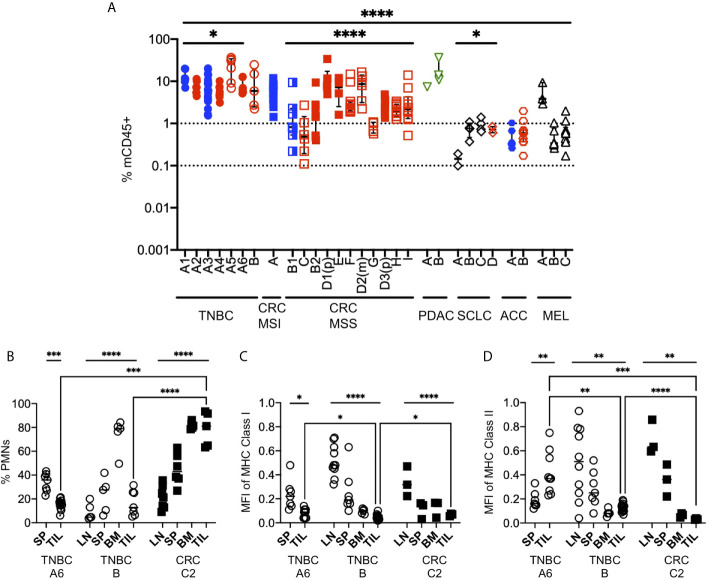
Mouse myeloid cells in human tumors of HIS-BRGS mice. **(A)** Infiltration of mCD45^+^ cells into human PDX or CDX in HIS-BRGS mice, same tumors as for **Figure 4B**. *p < 0.05, ****p < 0.0001 [Welch’s ANOVA among means within experiments with same tumor type or across tumor types, lines indicated the tumors included in the analysis] **(B)** Frequencies of mouse neutrophils (PMNs) in lymph tissues and human tumors (TNBC, CRC). Normalized expression (geometric mean fluorescence intensity) of mouse MHC Class I **(C)** and MHC Class II **(D)** expression in total mCD45^+^ cells. Expression was normalized to mCD45^+^ positive control cells for each day for comparison. *p < 0.05, **p < 0.01,***p < 0.001, ****p < 0.0001 [(**B–D**: Upper lines show two-group t test or Welch’s ANOVA among means of different organs in the same experiment. Two-group t-test with Welch’s correction were performed between the means of TILs (brackets)].

Mouse immune cells, which are strictly of the myeloid lineage due to the absence of T, B, NK and ILC lymphocyte populations, have been often ignored in HIS-mouse studies, with few exceptions (99). However, they represent the dominant myeloid population and therefore likely play a role in Ag presentation to the HIS and in TME immunomodulation. As with other immune cell types, myeloid cells are composed of multiple sub-types, with immunostimulatory or inhibitory functions (100, 101). We enumerated the frequency of mCD45^+^ cells in tumors excised from HIS-BRGS mice with flow cytometry. We observed more mCD45^+^ cells than hCD45^+^ cells, indicating a predominant myeloid rather than lymphocytic infiltration in tumors. In addition, we observed a narrower range of mCD45^+^ infiltrating cells than hCD45^+^ cells across all tumor types (**Figure 9A** compared to **Figure 4B**, mCD45+ range 0.1-100, hCD45+ range 0.001-100). The variability was similar for both hCD45+ and mCD45+ cell infiltrations into the TNBC A1-A6 experiments (p=0.022, Welch’s ANOVA) but higher variability was observed among the CRC MSS PDX models (p=0.0028 for hCD45+, p<0.0001 for mCD45+, Welch’s ANOVA), suggesting differences among PDX TMEs. We found much higher infiltration of mCD45^+^ cells in the PDAC and some CRC models, tumors that are resistant to immunotherapy and may contain a dominant myeloid immunosuppressive population, represented by mCD45^+^ cells in our model. On the other hand, the Lynch syndrome-associated ACC PDX “A2” had a higher ratio of hCD45^+^/mCD45^+^ cells relative to other tumors (**Figure 9A**). The decreased mCD45^+^ presence may be relevant to the positive ICB immunotherapy response in this model (48).

In our experience, we routinely notice two distinct populations of mouse cells: a mCD45^hi^ and a mCD45^lo^. We stained the mouse cells with GR1, F4/80 and Ly6G to distinguish granulocytes (polymorphonuclear, PMNs), defined as mCD45(hi)GR1^+^F4/80^-^or Ly6G^+^ from macrophage lineage, mCD45(int)GR1^-^F4/80^+^Ly6G^-^ (**Figure 9B**). We found that neutrophils were a minor population in the LNs, intermediate in the spleen but dominant in the bone marrow, consistent with physiological distribution in mice or humans. However, the frequency of mouse neutrophils in the tumors were tumor-dependent, with very high populations in a non-responsive CRC and low frequencies in ICB-responsive TNBC. We next queried whether the expression of mouse MHC molecules was altered in tumors of these mice, as is common for myeloid TILs. We stained the mCD45+ cells for expression of mouse MHC Class I (anti-H-2) and MHC Class II (anti-I-A/I-E) (**Figures 9C, D**). We found that the expression of mouse MHC molecules was highest in the LNs and was actually downregulated in the spleens and, even more so, in the bone marrow of HIS mice. In all cases the expression was lower (ratio <1.0) than that from a wild-type mouse, which we used as normalization control. Furthermore, the expression of MHC class I was lowest in the TILs of the mouse, regardless of tumor type (TNBC or CRC, **Figure 9C**). Similar to MHC Class I, MHC Class II expression was lower in TILs of both the TNBC PDX and CRC PDX mice; however, it was higher in the TNBC CDX tumor relative to the spleen (**Figure 9D**). Together, these data support a likely immunosuppressive role of the mouse CD45^+^ cells in the TME of the implanted tumors.

## Discussion

Here we present our studies testing human immune responses to human tumors, in our HIS-BRGS mouse model. The model is powerful in its ability to test a variety of tumors (either CDX or PDX), each harboring their own individual genotypes and corresponding mechanisms of immune evasion. The acceptance of these tumor allografts in the presence of a HIS with both innate and adaptive arms of the immune system represents a central paradox of this model. These tumors are immunologically allogeneic to the HIS that has developed in the murine host from a non-HLA matched HSC donor (CB or fetal liver in most cases, as adult tissues yield few CD34^+^ cells) (61). We consider this the beauty (the coveted ability to study human tumors in the presence of a HIS in a small animal model) and the beast (the lack of rejection of the allogeneic tumors) of the system. Thus, a significant question remains to be solved: why does the human immune system in these mice not reject the human tumors? In our preliminary data, we found that tumors can grow if implanted early or late after engraftment, i.e. in the presence of human T cells. The tumor acceptance suggests that the HIS in these mice is impaired and incapable of mounting an adaptive immune response to reject the tumor. This response could be explained by 1) an immature, uncoordinated immune system that lacks proper human-specific cytokines, chemokines and receptor interactions, or 2) a highly activated and immunosuppressed immune system. Several publications reveal the lack of robust T cell dependent immune responses to immunological challenges in HIS mice (36, 51). B cells are also reported to not fully develop (43, 102), although in older HIS-mice, mature B cells are found in the spleen, and are abundant in the LNs (56). Histological analyses show suboptimal TB coordination, typically associated with poorly formed germinal centers (43, 56). However, in our studies of HIS-mice spanning more than a decade, we find signs of robust, yet admittedly still incomplete, HIS responses if evaluated months after engraftment (56).

We present data supporting that this lack of allograft rejection is due in part to the immunosuppressed nature of the HIS in these mouse models, and not merely an immature immune system. Many T cells in HIS-mice are of the memory phenotype, more like a human adult than a newborn (56, 103). This activation appears to be Ag-driven and not merely the result of lymphopenia-induced activation as 1) it occurs months after engraftment, and 2) the presence of many CD28^-^ T cells, which are the result of late-stage Ag-activation (data not shown) (103). Notably, both CD4^+^ and CD8^+^ T cells are highly activated and express abundant inhibitory receptors such as PD-1 and TIGIT, yet less TIM-3. We have observed an increase of exhausted T cells over time with increased PD-1 expression with age (23). Importantly, the ability of the T cells to recover from exhaustion has been shown through administration of anti-PD-1 Abs that have correlated with reduced tumor burden for certain human tumors and even EBV-induced lymphomas (23, 48, 50, 104, 105). This feature suggests that a mature HIS can develop in these mice with the ability to mount adaptive immune responses; however, exhaustion must first be overcome. The exact nature of this immunosuppression in HIS mice is still unknown, although it likely relates to the xenogeneic nature of thymic selection.

We conclude that tumor immunotherapy studies in these HIS-mouse models are best suited to test the ability of a treatment to inflame a tumor. The treatment must also release the inherent immunosuppression in the HIS-mouse. Tumor growth inhibition and changes in immune responses in the tumors represent the experimental readouts. However, the validation of this model relies on its’ ability to distinguish the release of immunosuppression among therapies and individual human tumors, i.e. if all tumors respond equivalently the model is not useful.

### Immune Responses to Individual Human Tumors Are Unique in HIS-BRGS Mice

Tumors evolve distinct mechanisms of immune evasion, including limited access of the immune system to the tumor, accumulation of immunomodulating cells, or increase in immunosuppressive molecules (12). A valid representation of a TME is essential to an immunotherapy-based preclinical model and is the appeal of any *in vivo* setting, as the TME is very difficult to truly replicate *in vitro* (16, 17, 31). Here, we present flow cytometry data showing human cell infiltration varies by tumor type. This is similar to studies evaluating HIS infiltration by IHC or flow cytometry in the same (44) or a similar (84) model. In all cases, the infiltration of the human lymphocytes is lower than that of the mCD45^+^ myeloid cells, a situation similar to the accumulation of myeloid lineage cells in human tumors. However, in most cases the relative patterns of mCD45+ infiltration into individual tumors mirrored that of the hCD45^+^ cells, supporting a tumor-dependent influence of immune infiltration *in vivo*. We have observed a few examples supporting that some ICB-responsive tumors have increased infiltration in our model (TNBC A1-6 and Lynch syndrome-associated ACC A) (23, 48). Furthermore, three SCLC tumors appeared “cold” with very little infiltration, even mCD45+ cells, a phenotype consistent with their limited response to ICB treatments in clinical trials (106). We also demonstrated more reproducible hCD45^+^ and mCD45+ infiltration in tumors from the same origin (e.g., TNBC A1-6) compared to tumors from distinct PDXs (e.g. CRC MSS), that showed higher variability in immune cell infiltrations, reflecting the unique phenotype of each tumor. The differences in immune infiltration observed in repeat experiments (e.g. TNBC A1-A6, CRC D1P/D3P) are small compared to differences in average infiltrations across all tumors (p<0.0001); however, these differences do reflect the limitations of the model. We suggest that differences in human chimerism, CB donor genetics or changes in the tumor characteristics with passages in culture or mice are contributing factors to this variability. More replicates with different batches of HIS-mice are necessary for confirmation. Collectively these data support the ability of HIS-mouse models to represent the unique properties of individual human tumors.

The results of our study suggest monitoring the immune response may be more informative, or at least complementary, than the actual tumor growth in HIS-BRGS oncology models. Although we achieved high tumor take rates for most models, we observed an effect of chimerism on the growth of individual tumors, even in the absence of treatments. The presence at end of study of cytotoxic T cells in the lymph organs of mice with slow-growing tumors supports a role of the HIS in controlling the allogeneic tumor growth. Thus, tumor growth curves might be influenced by HIS responses in the untreated group.

On the other hand, we consistently detect changes in individual immune parameters among treatment cohorts, even with similar average growth rates. The differences among groups show statistical significance even in experiments with as few as 3 or 4 HIS-mice per group (i.e. typically>5 tumors). Furthermore, when the immune parameters were correlated to the SGR, we did observe the tumor size as a function of the immune response, e.g. smaller tumors appeared to be enriched in CD4^+^HLA-DR^+^ T cells in HIS-CRC-BRGS mice treated with combination immunotherapy. This correlation held up over 5 independent CRC PDXs treated with the same drug combination, although to varying degrees. Our studies testing another combination immunotherapy for CRC MSS highlight the influence of individual tumors on immune responses, as seen in distinct GrB^+^ T_EM_ CD8^+^ versus CD4^+^TNFα^+^ driven responses to another tumor (CRC H,I; **Figure 7C**). These mice were generated from the same CB, and injected one week apart, leaving the PDX as the largest experimental variable in this case. To further illustrate this point, we have previously reported an increased CD8^+^IFNγ^+^ signature upon treatment with anti-PD-1 Ab (nivolumab) to both a TNBC CDX and CRC MSI-H PDX, similar to reports from studies in mouse models or human trials (23, 73, 91, 95, 107). In another study, increased CD8^+^GrB^+^TIM-3^+^CD28^-^ cells correlated with smaller ACC tumors that had been treated with pembrolizumab, also an anti-PD-1 Ab (48). It is interesting to note that we can observe either or both CD4^+^ or CD8^+^ T cell responses in different scenarios. The importance of CD4^+^ T cells in a tumor immune response is gaining acceptance (108), and clinical trials and syngeneic mouse model studies have similarly identified distinct immune correlates (53, 54, 94, 109, 110). As in clinical trial studies, these data are strictly correlative. In our studies, the immune correlates are most significant among human T cells in the TILs, with a few exceptions in which we have observed systemic treatment effects in either the LN or spleen. The ability to test the responses in secondary lymph organs, and even more importantly in the tumors, is a major advantage of this model. In clinical trials, the amount of tumor tissue is often limited to less-quantitative IHC analyses while the systemic immune system is limited to the blood.

A further advantage of the HIS-model for immunotherapy studies is the ability to evaluate the cancer cells themselves. We have detected increases in human HLA molecules and changes in inhibitory receptors such as PD-L1, that are consistent with increased IFNγ responses. Integrating these data with spatial information from IHC (or other techniques) can provide valuable information on cell interactions within the TME, as we have previously reported (23, 48).

All-in-all, this study supports the use of HIS-BRGS model to evaluate immune responses to distinct therapies for a wide repertoire of individual tumors. In this model, the activation of the T cells requires release of the inherent immunosuppression, a condition that may mirror the suppressed immune system in a tumor.

### Considerations for Experimental Design Using HIS-CB-Mouse Models

We acknowledge the limitations of the model and strongly advise consideration of engraftment and reconstitution details, particularly the late appearance of human T cells and LNs. In our hands, the model has an optimal experimental window, typically between 18 and 30 weeks of age, to test T-cell directed immunotherapies. Therefore, timing of tumor injections is critical and should be designed to insure 1) adequate T cells (typically >18 weeks in our lab) at time of treatment, and 2) a multi-lineage immune system, as B cell and myeloid populations are less frequent in older mice (typically >30 weeks). Each laboratory should test their own kinetics and long-term engraftment, as they may differ due to model and experimental protocols. For xenografts with slow growth rates, we suggest implanting early so they establish at around 18 weeks of age. This often requires testing growth rates in immunodeficient mice prior to use in HIS-mice. We have also shown that the human T cell chimerism affects the degree of human immune infiltration into tumors, and even limited control of tumor growth by the HIS in the absence of therapies. Thus, it is important to control for this variability in experimental design: our approach is to allocate mice into equivalent groups based on human, T cell, and CD8 frequencies. More recently, we have used the matched animal analysis (MANILA) software for random group allocation and treatment assignment, guaranteeing balanced interventions according to these parameters (111). The generation of HIS-mice from distinct CB units is another source of variability that should be considered. As in humans, but not syngeneic mouse models, the immune system in HIS-mice is genetically unique. However, unlike humans, the environment of these mice is well controlled in terms of age and immune exposures. Wang et al. analyzed studies in HIS-mice prepared from different CBs, but injected with the same tumors (50). They concluded that CB genetics were responsible for differences in tumor growth outcomes in these studies, although they did not test the influence of human chimerism. They also determined that the HLA match of the CB to the tumor had little contribution, not surprising since there was a maximum of 4 HLA matches, leaving a large alloreactive T cell repertoire (50). Although we have not formally addressed this parameter, we provided examples illustrating differences in immune responses to unique CRC or DLBCL tumors in our HIS-BRGS models that were treated with the same therapies in mice generated from the same CB. In conclusion, our numerous studies testing immunotherapy treatments on human tumors in HIS-BRGS-mice highlight the inherent variability in the system that includes: 1) age of mice and corresponding immune system (T cells develop late), 2) human chimerism, 3) CB donor and 4) the human tumor. Our experimental approach strives to minimize these variables, stressing the differences in human tumors.

Another important consideration in any study of immune correlates to an immunological challenge (tumor, pathogen, vaccine) is the kinetics of the response. Each data point represents a snapshot in time of the immune response. It is well appreciated that this response is a dynamic and complex process that includes a typical early activation event. The fate of individual cells is unique, as some will become non-functional or die, some will become memory, and some will proliferate and become effector cells, secreting a variety of cytokines. On a single-cell level, changes in activating and inhibitory receptors ensue along with transcriptional and epigenetic reprogramming. Analyses of immune correlates at a single point in time, whether on a single cell or a population level, provide an “aerial” view of the immune system. In our model, the difference in tumor growth rates and the work required on harvest day, limits our analyses to typically 4-8 mice on a single day. We have the added challenge that the immune system in the mice changes over time. Therefore, it is likely that our observed immune responses may be impacted by different timepoints: for example, more TIM-3 expression may represent a more exhausted response (i.e. TIM-3 is upregulated after PD-1 in an activated T cell). We do attempt to analyze similar numbers of mice from each experimental cohort on each day.

### Flexibility of HIS-Mouse Models

Immunotherapy research and technology advance at a brisk rate and preclinical models must follow the pace. This HIS-mouse model offers the opportunity for in-depth mechanistic study of the kinetics of anti-tumor immune responses. Given that we can generate 60 HIS-mice from the same CB, implant the same tumor, and evaluate both lymph tissues and tumors from the same mouse, experiments can be designed to evaluate the response in cohorts from various interventions, at defined time points. We have done this in a limited fashion with a nivolumab treatment of MDA-MB-231 CDXs and found that the response is indeed distinct over a 10-day period, with a significant increase of CD8^+^IFNγ^+^ T cells observed only after 21 days of treatment (23). The model offers the flexibility to similarly test dosing regimens. Single cell flow, mass cytometry, RNAseq, and ATACseq techniques can be utilized for in-depth immune and tumor characterization as well. Depletions of immune cell populations, such as CD8^+^ T cells, have also been performed in these HIS-models, to garner mechanistic information concerning the roles of distinct immune populations (50). An additional approach with the HIS-mouse model is the ability to genetically manipulate the HSC prior to “humanization,” and thus track a tumor-specific T cell receptor (112) or test the role of distinct genes in a response (e.g. knock-out IFNγ). Likewise, a human CDX can be genetically manipulated to test the role of a cancer gene. In addition to ICB studies, HIS-mice have been used to test CAR T cells (113). We propose this model should support testing a range of immune-related therapies.

Thus, the HIS-models offer robust flexibility to study human immune responses to human tumors in a preclinical *in vivo* setting. It is important to note that a small animal model in a controlled facility will always have much less variability than a human trial. Furthermore, experimental replicates between treated and untreated animals are possible and provide increased experimental power. These studies in the HIS-model are limited mainly by their relative expense and technical challenge. In comparison to syngeneic mouse models, these mice are labor-intensive and require expertise in their use and evaluation. However, they are a fraction of the cost of a clinical trial. On the other hand, organoids represent an improved 3-D *in vitro* model for testing combination immunotherapies (114). As with any model, this model has advantages and disadvantages in its representation of the TME (115, 116). The ultimate validation as preclinical models for the testing of immunotherapy regimens is correlation to the success in the clinic. We have presented limited data showing clinical correlation: 1) a case study of a patient with ACC responding to pembrolizumab in the clinic and an immune response in a matched PDX in our HIS-BRGS mice (48), and 2) correlation of increased immune response and reduced tumor growth in nivolumab-treated CRC MSI-H PDX compared to CRC MSS PDXs, consistent with clinical trial data. We are currently testing an immunotherapy combination in our model on PDXs derived from patients in a clinical trial testing the same drug regimen. These correlations with clinical trials are necessary for true validation of the HIS mouse model for these studies.

Immune-related adverse events represent significant ICB-associated toxicities in the clinic (117). Indeed, HIS-mice have been reported to show indications of these toxicities upon administration of human ICB-drugs (118, 119). We are currently also testing the efficacy of the HIS-BRGS mice as a preclinical model for testing ICB-related toxicities in the presence or absence of a human tumor. In all experiments, we measure animal weights along with tumors and do observe significant loss of weight in very few mice and this does not correlate with treatment. We have observed occasional instances of ICB-related colitis/diarrhea and salivary gland changes in our experiments with this model; however, these are not in the majority of animals. The model offers the ability to analyze a wide array of tissues, to evaluate human immune infiltration by IHC, as an indication of autoimmune-infiltration. In our model, the HIS system is tolerant of mouse tissues due to its development and selection in the mouse thymus. Thus, the allo-response should be directed specifically to the allogeneic tumor upon release of immunosuppression. Immune reactivity to the mouse tissues would represent autoimmune responses.

### Improvements to HIS-Mouse Models

As with all experimental models, HIS-mice harbor unique limitations that must be considered in the data interpretation. Specifically, the myeloid population is more variable in its chimerism than either the T or B lymphocyte populations in “basic” HIS-mouse models (67, 68, 120). We have found that their presence is more pronounced in tissues and indeed we have observed a much higher frequency of myeloid cells in tumor than in either LN or spleen (23, 48). To address this relative human myeloid deficiency in HIS-mice, several groups have generated immunodeficient recipients with transgenic human myeloid specific cytokines, including the NOD-scid IL2Rγnull-3/GM/SF (NSGS) (121) and MISTRG (68) models. Human tumors grow in NSGS mice and therefore this model has also been used for immunotherapy preclinical studies (122). The MISTRG model is best equipped to grow myeloid marker specific tumors such as Acute Myeloid Leukemia (123). However, there have been reports of short experimental windows and anemia associated with these models (69, 124). Alternative models to improve human myeloid reconstitution are immunodeficient mice harboring a mutation in the c-kit gene (NSGW41 or NBSGW) (125, 126). This mutation interferes with mouse myeloid production and allows increased human myelopoiesis due to lack of competition. Additional advantages to these models are the lack of irradiation required for engraftment, increased human erythropoiesis and increased HSC longevity in the mouse (99, 103, 124). We are in the process of testing the HIS-NBSGW strain for immunotherapy studies in an effort to develop a more representative HIS-mouse model with reduced myeloid mouse-to-mouse variability.

An additional and important limitation is the allogeneic nature of the implanted tumor. In the “basic” HIS-models T cell selection occurs typically on mouse MHC molecules (103, 127). Significant efforts have been made to include human MHC molecules in HIS-mice, in order to study Ag-specific responses. One approach has been incorporating human HLA transgenes in the recipient. The HLA-A2 NSG mouse recipient has been the most widely used of these models, due to the high representation of this allele in the general population (127, 128). One can thus select HLA-A2^+^ CB or fetal liver samples to generate the HIS-mouse, and HLA-A2^+^ tumors. Although this approach includes some tumor specific reactivities, there is still overwhelming alloreactivity to other HLA molecules, since T cells will also be selected on other mouse MHC molecules and react to the non-HLA-A2 human alleles. Another approach is to “knock-out” the mouse MHC genes and force selection exclusively on the human HLA (129). Hu et al. also transfected the HSC with a tumor-specific T cell receptor transgene prior to humanization. This system enabled studies of a human T cell response to a MART tumor-specific Ag (112). Although very relevant information can be garnered from this approach, tumor responses include multiple Ags of varying affinities (130). Furthermore, both CD4^+^ and CD8^+^ T cells influence the TME. Indeed, HIS-mice with both human MHC Class I and Class II transgenes showed greatly improved immune responses over either transgenic alone (131, 132).

Other groups have solved the T cell selection problem with the transplant of a thymus of fetal origin matched to the donor liver in the liver-thymus model (133). This system allows a complete histocompatibility match of the immune system and the thymus, especially if the mouse MHC is also absent. However, matching a tumor to this system has the same odds as a bone marrow transplantation, i.e. realistically improbable. Therefore, the tumor will still be allogeneic to the immune system. One must also keep in mind that a more functional HIS, as in both the liver-thymus and human HLA-transgenic mouse models, should react better to a human tumor that is not completely HLA-matched. Thus, the essential feature of growing a human tumor in a HIS-model may be compromised in these models. There are few reports of immunotherapy studies in either of these models, which may be indicative of compromised multi-lineage tumor growth, a concept that needs to be formally assessed (122, 134). In the end, a practical HIS-model may require balancing the most representative HIS with reduced natural immunity (rejection) of the tumor.

The ultimate goal for a preclinical small animal model is to make a true “avatar”- a mouse with a HIS generated from the same patient as the tumor (45). This would also require a matched thymus or some source of T cell selection. Practically speaking we are years away from this goal: 1) adult HSCs in unmanipulated blood do not generate human chimerism in immunodeficient mice (57) and therefore the HSCs from the patient, who often harbor inferior stem cells due to the disease, must be acquired from bone marrow stem cells, a population that generates inferior HIS-mice in both quantity and quality (45, 61, 135); 2) a matched thymus is required for proper selection of the T cells - the surgical acquisition of a matched thymus, which in adults is not present due to involution, is an obvious barrier, leaving the technologically-challenging induction of a thymic organ using induced pluripotent stem cells as the best option; and finally 3) the timing of generating these chimeras with matched tumors/thymus/HIS takes many months (minimum 6), a period that is too long for a practical personalized-medicine approach. In the meantime, the HIS-mouse immediately offers a model capable of testing combination immunotherapies and interrogating the immune responses to unique human tumors. This work suggests caution in experimental design to achieve optimal data.

## Data Availability Statement

The original contributions presented in the study are included in the article/**Supplementary Material**. Further inquiries can be directed to the corresponding author.

## Ethics Statement

The animal studies were reviewed and approved by University of Colorado AMC Institutional Animal Care and Use Committee. All acquisition of human tumor samples were consented under IRB approvals as indicated in Materials and Methods.

## Author Contributions

JM-J, AC, and JL are responsible for the paper concept and overall experimental designs. JM-J, ML, and JL analyzed the data, wrote the manuscript, and prepared the figures. BF contributed acquisition of cord bloods. HY, CR, RT, ED, JES, RP, SGE, WM, MV, CL, JW, JRD, KK-V, and TP all contributed funding, experimental design and/or intellectual discussions. AC, ML, SB, SH, JS, NN, HY, CR, XW, JB, DG, AK, AY, DT, and JL all helped perform experiments in the HIS-BRGS mice. JS, JB, NN, ML, and JL generated and managed the humanized mouse colony. JES, RP, RT, TP, MV, JRD, and KK-V helped with editing of the manuscript. JM-J, JTD, and PB provided help with data analysis and statistics. All authors contributed to the article and approved the submitted version.

## Funding

This work was supported by NIH NAIAD RO1 (I124474 and AI124474S, RP/JL), NCI RO1 (R01CA229259-01, CL), CPRIT Scholar in Cancer Research grant (RR160093, SE), DOD Career Development Award (CA191245, AC), NIH-NCI K08 (K08CA222620, KK-V) and Cancer League of Colorado, Golfers Against Cancer Award (KK-V).

## Conflict of Interest

The authors declare that the research was conducted in the absence of any commercial or financial relationships that could be construed as a potential conflict of interest.

## Abbreviations

ACC, Adrenal adenocarcinoma; BRGS, BALB/c-Rag2^null^Il2rγ^null^SIRPα^NOD^; CB, Cord blood; CDX, Cell line-derived xenograft; CRC, Colorectal cancer; DLBCL, Diffuse Large B Cell Lymphoma; HIS, Human Immune System; HSC, Hematopoietic Stem Cell; ICB, Immune checkpoint blockade; ILC, Innate lymphoid cell; LN, Lymph nodes; MISTRG, M-CSFh, IL-3/GM-CSFh, hSIRPAtg; TPOh; Rag2-/-; IL2RgC-/-; NK, Natural Killer; NSG, NOD/SCID/Il2rγ-null; NSGS, NOD.Cg-Prkdcscid Il2rgtm1Wjl Tg (CMV-IL3,CSF2,KITLG)1Eav/MloySzJ; PBMCs, Peripheral blood mononuclear cells; PDAC, Pancreatic ductal adenocarcinoma; PDX, Patient-derived xenograft; SCLC, Small cell lung cancer; SGR, Specific growth rate; TIL, Tumor infiltrating leukocyte; TME, Tumor microenvironment; TNBC, Triple negative breast cancer.
