# Supplementary material for: Testing Cancer Immunotherapy in a Human Immune System Mouse Model: Correlating Treatment Responses to Human Chimerism, Therapeutic Variables and Immune Cell Phenotypes
Source: Front Immunol. 2021 Mar 29;12:607282. doi: 10.3389/fimmu.2021.607282 (PMC8040953; doi:10.3389/fimmu.2021.607282)
Supplement: Supplementary file 1 [file DataSheet_1.docx]

Supplementary Material

# Materials and Methods

**Stem Cell Isolation**

HSCs were isolated from PBMCs prepared from clinically-rejected CB units from the University of Colorado Cord Blood bank (Clinimmune Labs, Aurora, CO) using CD34+ magnetic Miltenyi beads, and expanded in short-term cultures with IL6 (10 ng/ml), SCF (40 ng/ml) and FLt3L (20 ng/ml). CD34+ cells, harvested between days 4-6, were frozen in 90% FCS/10%DMSO and stored at -80°C prior to injection into neonate pups. Investigators were blinded from donor identities and the studies were performed in compliance with University of Colorado Institutional Review boards (COMIRB#16-0541).

**Generation of HIS-BRGS mice and chimerism evaluation**

To generate human immune system mice, neonatal (d1-3) BRGS (BALB/c*Rag2*^nul^*^l^IL2Rg*^null^*Sirpa*^NOD^) pups, obtained from the laboratory of James Di Santo, were irradiated with 300 rads 2-6 hrs prior to injection with 0.2-0.6x10^6^ expanded then thawed CD34+ cells. The number of cells injected is equivalent to 50,000 fresh CD34+ cells per mouse, i.e. cell count prior to *in vitro* expansion. Mice were bred and engrafted in the University of Colorado Denver Anschutz Medical Campus vivarium with prior Institutional Animal Care and Use Committee (IACUC) protocol and in a facility accredited by the American Association for Accreditation of Laboratory Animal Care. BRGS mice, both breeders and engrafted, were maintained on an alternating bi-weekly Septra-enriched (Uniprim, Harlan) diet. Mice were injected in the facial vein, liver or both.

**Tissue Harvest and Processing**

At end of study, tumor-bearing HIS-BRGS-mice were euthanized via CO_2_ inhalation followed by exsanguination *via* cardiac puncture. The sera were separated using a microcentrifuge (4°C, 9000 rpm for 6’) and frozen for future analysis. Tumors, spleens, LNs (axillary, brachial, inguinal, cervical, pyloric and mesenteric), and in some experiments also bone marrow, liver, lung and gut, were excised. LNs and spleens were collected and processed into single-cell suspensions by shearing the tissue between frosted glass slides in harvest media (1X Iscove’s DMEM, 5% FBS, 1% Glutamax, 1% HEPES, 1% pen-strep, 100μg/ml DNase) and filtering through cotton plugged long-taper Pasteur pipettes. 22g needles were used to flush the femurs and dissociate the BM. Spleens and BM were treated with ACK lysing buffer (800ml H_2_O, 8.29g 0.15M NH_4_Cl, 1.0g 0.1M KHCO_3_, 200ul 0.5M EDTA, pH 7.2-7.4) to remove red blood cells. Liver tissue was processed into single cell suspensions using chilled RPMI+10% FBS and mechanical disruption in a petri dish. Fat was removed and the tissue was passed through a 70μm filter in 9mL of media. Cells were isolated using ficoll-paque density gradient centrifugation and the mononuclear cell layer was collected. Both lung and intestines were collected in chilled RPMI + 10% FBS. Prior to processing, intestines were cut into ~2cm long pieces and all fecal materials and mucus were removed mechanically and by flushing with PBS. Both lung and intestines were digested using Liberase TL (25μg/mL) (Roche) and DNase I (50μg/mL) in a 50mL tube for 20’ (intestine) and 30’ (lung) in a shaking incubator at 37°C. After digestion, 10mL of chilled RPMI+10% FBS was used to pass through a 70μm filter, then centrifuged at 500xg for 5’. Supernatant was removed, and the cell pellet was resuspended in 9mL of PBS. Leukocytes were then isolated using ficoll-paque density centrifugation. The tumors were weighed upon excision, 1/8 was removed and frozen in liquid N_2_, while another 1/8 was submerged in 10% formalin for IHC imaging. The remaining ¾ tumor was lightly minced with scalpel or razor blade in serum-free media (1X RPMI-1640, 1% pen-strep, 1% HEPES, 100μg/ml DNase), 50μg/ml Liberase DL (25μg/mL) was added and digested for 40’ at 37°C on Miltenyi GentleMACS Dissociator. Soft tumors were digested using the hTDK1 and hard tumors were digested using the hTDK3 Miltenyi preset program. The tumor suspension was passed through a 100μm filter, which was then washed with 10ml of complete media (1X RPMI-1640, 10% FBS, 1% non-essential amino acids, 1% pen-strep, 1% HEPES, 100μg/ml DNase), pelleted at 1250 rpm for 10min. All single cell suspensions were resuspended in harvest media.

**Cell staining and flow cytometry**

Live/dead discrimination was performed with Zombie Dyes (Biolegend) in PBS at RT for at least 15’. For surface staining, single-cell suspensions were incubated with fluorescent-labeled Abs (Supplemental Table I) for 15 min at 4°C in staining buffer (1X PBS, 1% bovine serum albumin, 0.1% NaN_3_) and washed twice in staining buffer. All staining was performed in the presence of FcR Block (human, Miltenyi) and 24.G2 (anti-mouse CD32) blocking agents. The first stain was performed to assess human chimerism, T, B and myeloid cell populations. Down-stream intracellular staining and functional assays were performed only on samples with >100 T cells. To assess T cell cytokine (TNFα, IFNγ) production, approximately 2 million isolated cells from tumors and spleens were cultured overnight at 37°C and 5% CO_2_ in complete media (1X RPMI-1640, 10% FBS, 1% non-essential amino acids, 1% pen-strep, 1% HEPES, 100μg/ml DNase) supplemented with cell-stimulation cocktail (Invitrogen). To allow for accumulation of secreted proteins, Golgi-stop (BD Biosciences) was added for the final 4 hours of incubation. Adult human PBMCs with and without cell-stimulation cocktail served as internal technical controls within each plate. Intracellular staining was performed to visualize expression of TNFα and IFNγ (with cell-stimulation), or FoxP3, Tbet, Eomes and Granzyme B (directly *ex vivo*). First, cells were fixed in 1% para-formaldehyde in staining buffer at RT for 30’. Cells were then permeabilized with 0.5% saponin in staining buffer (TNFα, IFNγ) or 1X Perm Buffer (Granzyme B, FoxP3, Tbet, Eomes; Biolegend Transcription Factor kit) for 15’ at RT, followed by 30min incubation with fluorescent-labeled Abs. The cells were subsequently washed three times in appropriate permeabilization buffer, then twice more in staining buffer. The samples were resuspended in defined volumes of staining buffer and data was acquired on Propel Labs Yeti (BioRad ZE5) or Beckman Coulter Cyan flow cytometer, at a constant rate for an absolute volume, at the CU Cancer Center Flow Cytometry Shared Resource. Data was analyzed with FlowJo software (BD).

These panels were determined following numerous trials in which we excluded analyses of the following markers: CD69 and CD95, which were uniformly high in TILs and did not differ among treatment groups; Tbet which correlated well with IFNγ as expected; and Lag3, which we did not detect well on the cell surface likely due to poor anti-human Ab quality (data not shown)*.*

**ELISA**

Human IgM and IgG were measured in the sera of HIS-BRGS mice using sandwich ELISAs. To avoid detection of immunotherapy drugs (human IgG), we used monoclonal anti-human IgM and IgG to measure total hIg concentrations. Plates were coated with mouse anti-hIgM (SA-DA4) or hIgG (H2) in PBS and incubated overnight at 4°C. Plates were washed four times in PBS w/ 0.1% Tween then blocked for 2hr at RT in staining buffer and washed four more times. Sera were diluted 1:10 in staining buffer then added to plates in two-fold serial dilutions. Standard hIgM or hIgG was added to plates in duplicate rows at threefold serial dilutions starting at 5 µg/ml. Plates were incubated overnight at 4°C and washed four times in the morning. Anti-hIgM (SA-DA4) or hIgG (JDC-10) secondary alkaline phosphatase (AP)–conjugated Abs were then added at 1:500 dilution and incubated for 2hr at 37°C. The plates were washed four times before adding developing buffer (AP substrate p-nitrophenyl phosphate, Sigma-Aldrich) solution. Absorbance was measured at OD_405_ on a VersaMax plate reader (Molecular Devices) at regular intervals.
