# Supplementary material for: Testing Cancer Immunotherapy in a Human Immune System Mouse Model: Correlating Treatment Responses to Human Chimerism, Therapeutic Variables and Immune Cell Phenotypes
Source: Front Immunol. 2021 Mar 29;12:607282. doi: 10.3389/fimmu.2021.607282 (PMC8040953; doi:10.3389/fimmu.2021.607282)
Supplement: Supplementary file 2 [file DataSheet_2.pdf]

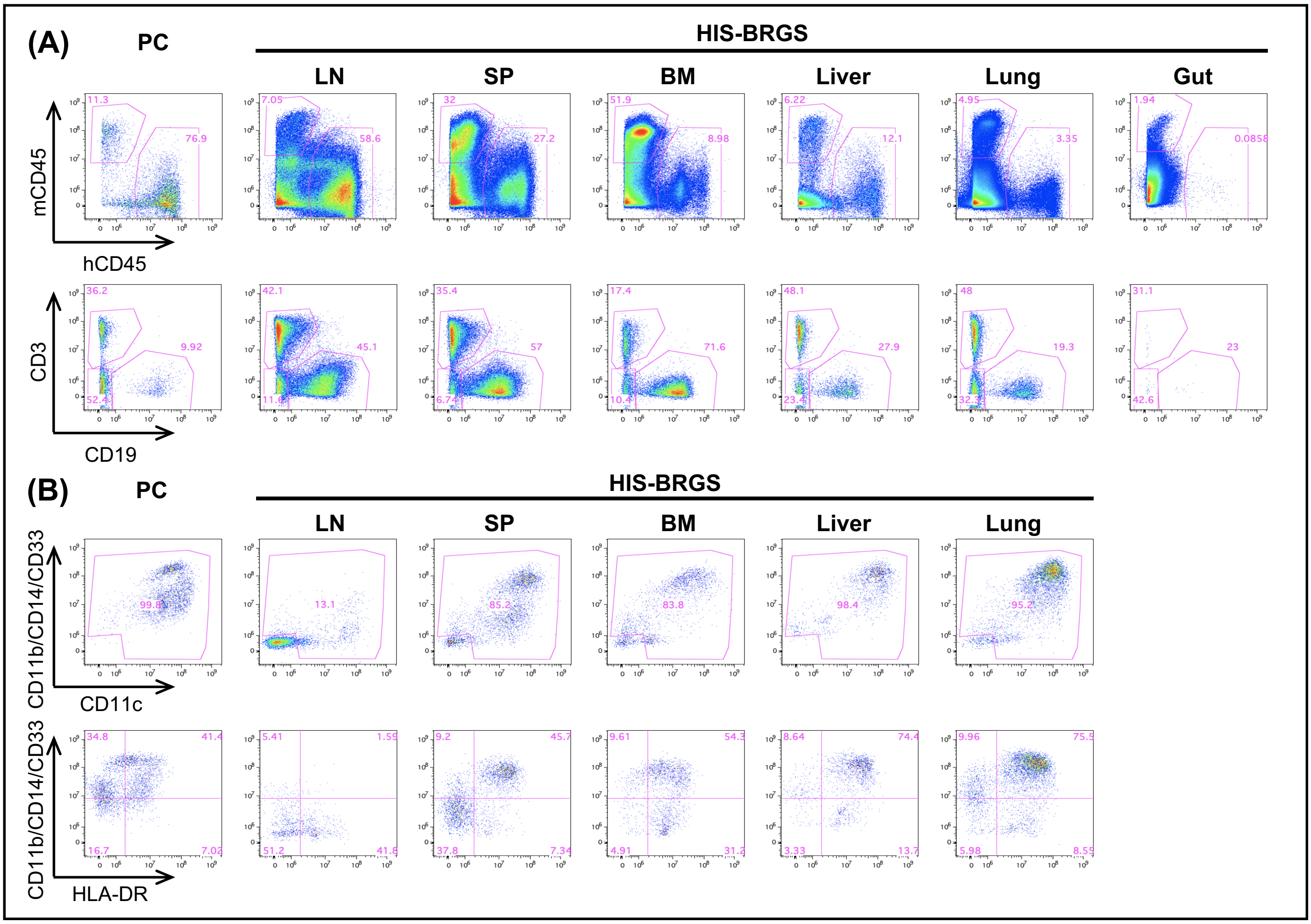

Supplementary Figure 1. (**A**) Representative flow analysis of human (hCD45+, top row), and T (CD3+) and B (CD19+) cells (bottom row, of hCD45+) in HIS-BRGS organs. Gate: single cells. (**B**) Myeloid populations (CD11b+, CD14+, CD33+ or CD11c+, top row of hCD45+) in HIS-BRGS organs. Expression of HLA class II molecules (HLA-DR) on the myeloid cell subset (bottom row). Gate: hCD45+CD3-CD19-. For (A) and (B), human PBMCs mixed with mouse spleen cells served as a technical staining control ("Pos Con", left column).

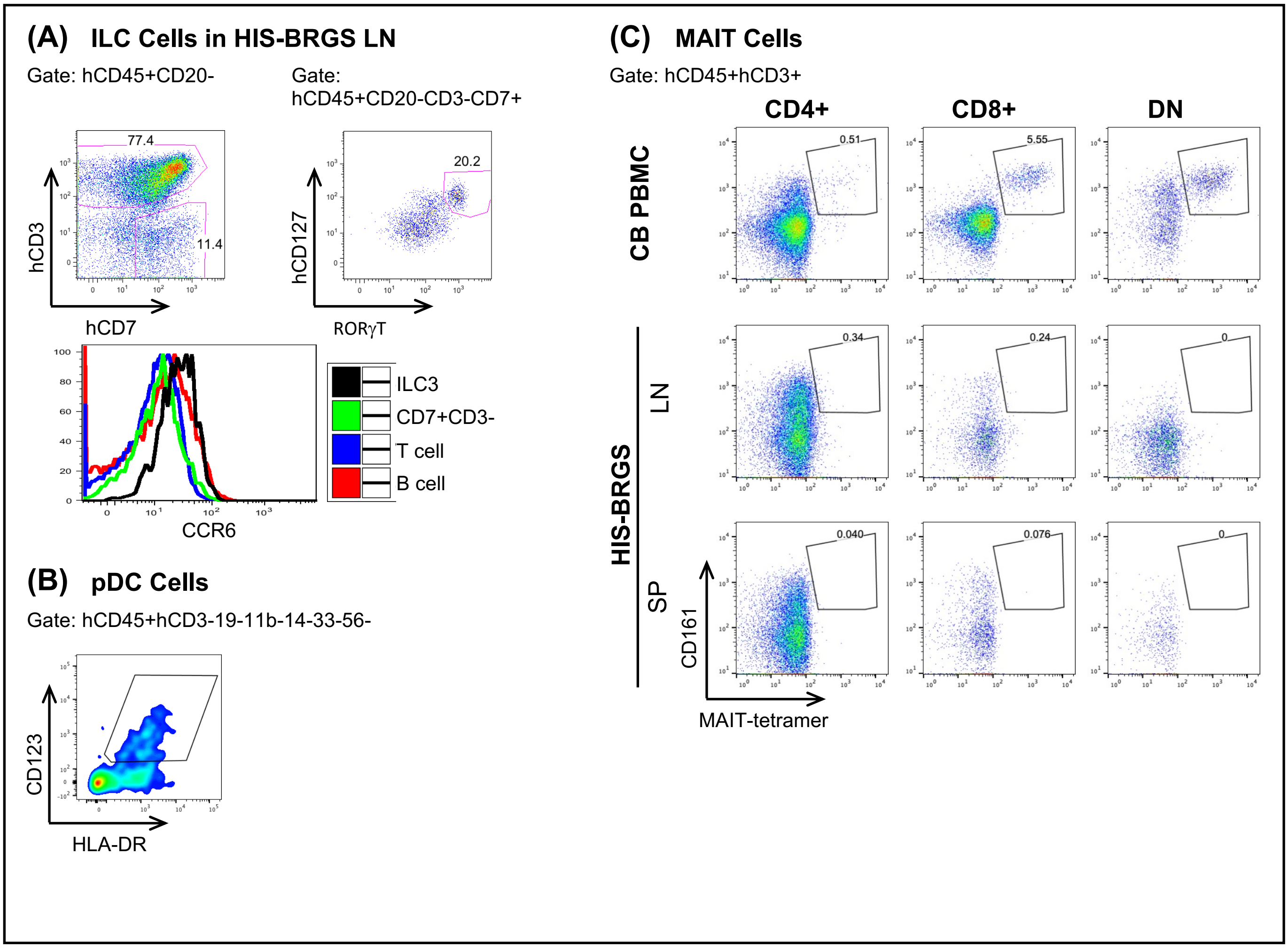

Supplementary Figure 2. Minority immune subpopulations in HIS-BRGS mice. Representative staining of **(A)** Innate Lymphoid Cells (CD7+CD3-,hCD127+RORγT+CCR6+) and **(B)** plasmacytoid dendritic cells (pDC:Lin-HLA-DR+CD123+) detected in LNs of HIS-BRGS mice. **(C)** Mucosal-associated invariant T (MAIT) cells were not detected in any of the lymphoid organs of HIS-BRGS mice; PBMCs from CB served as positive staining control.

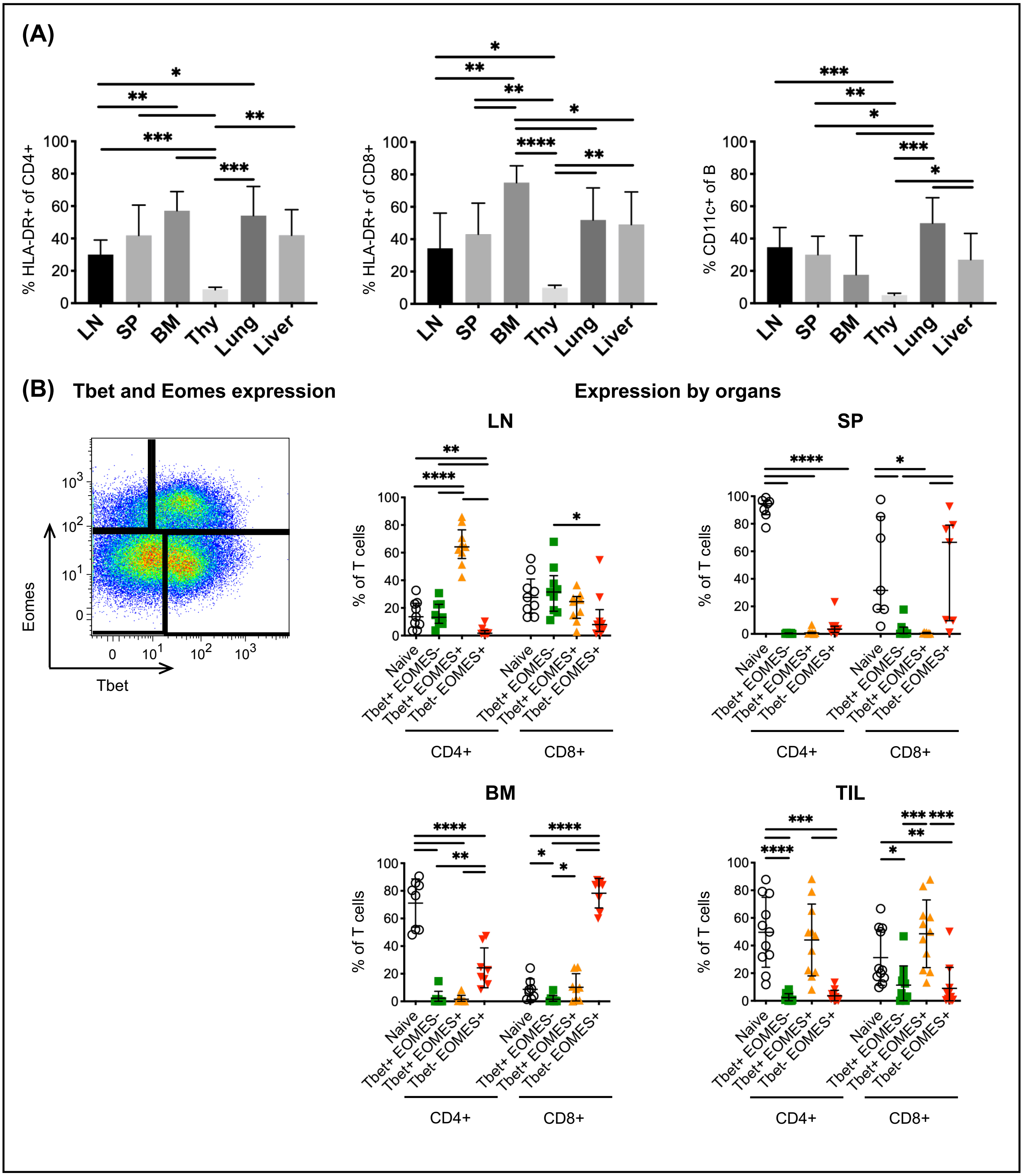

Supplementary Figure 3. Activated and exhausted lymphocytes in HIS-BRGS mice. **(A)** HLA-DR expression on CD4+ (left) and CD8+ (middle) T cells and CD11c+ expression on B cells (right) in different organs of HIS-BRGS mice. **(B)** Representative flow cytometric analyses of T-box transcription factors T-bet and Eomesodermin (Eomes) expression in the LN T cells of HIS-BRGS (left). Differential expression patterns of Tbet/Eomes transcription factors in CD4+ and CD8+ T cells in lymph organs and TILs (TNBC) of HIS-BRGS mice (right). *$p<0.05$, **$p<0.01$, ***$p<0.001$, ****$p<0.0001$ [Welch's t-test, two-tailed comparison as indicated by edges of each line].

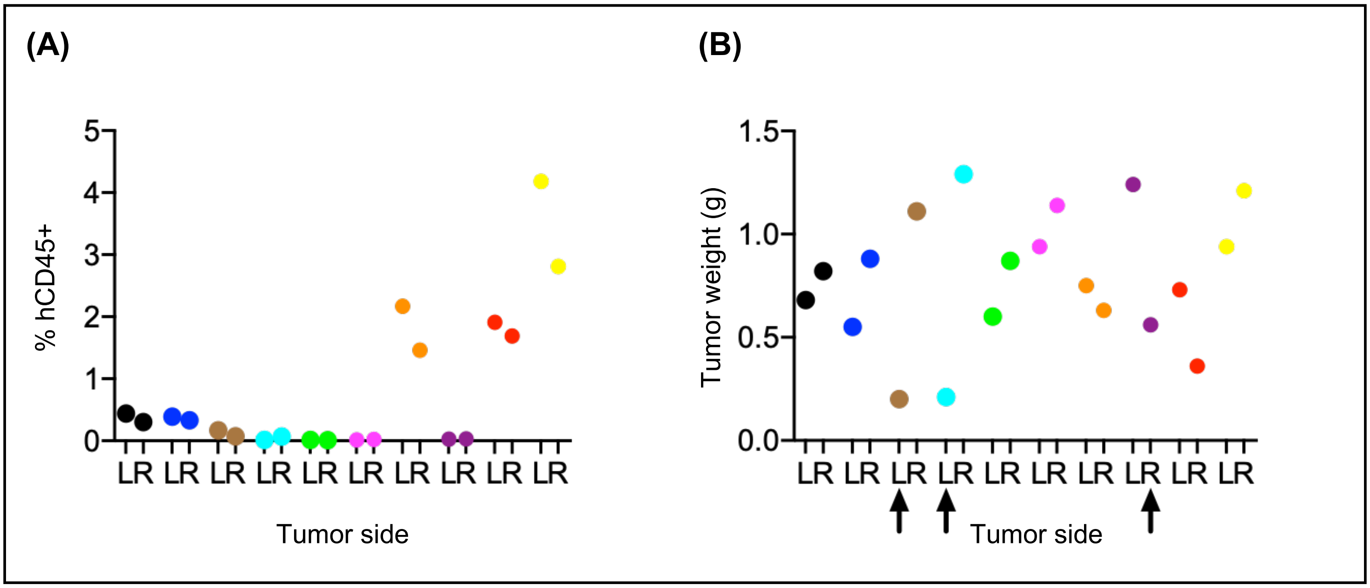

Supplementary Figure 4. Comparison of CRC tumors in left and right flanks of individual HIS-BRGS mice. Human CD45+ infiltration **(A)** and tumor weights **(B)** by left (L) and right (R) tumors in the same mouse. Each pair of symbols of the same color correspond to a single mouse.

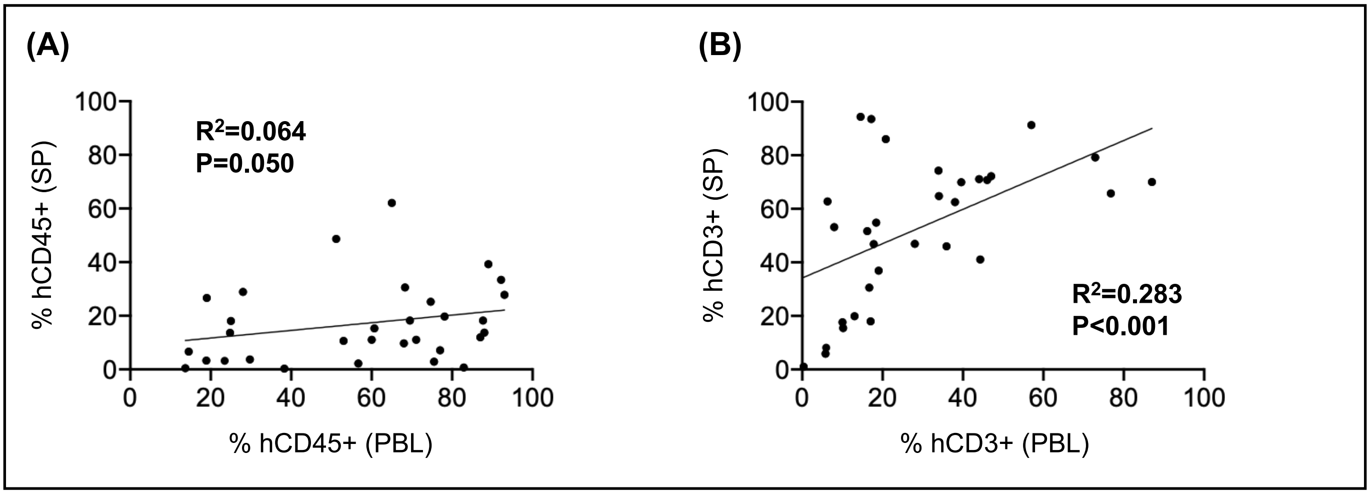

Supplementary Figure 5. Correlation of human chimerism in blood, measured prior to tumor injections, and in the spleen at end of study, weeks later**. (A)**: human (hCD45+) chimerism; **(B)**: T (CD3+) chimerism. Pooled data from 5 experiments of HIS-BRGS untreated mice implanted with TNBC MDA-MB-231. [Linear regression analysis, R-squared score (R^2) and P value (P) in bold if statistically significant ($P<0.05$)].

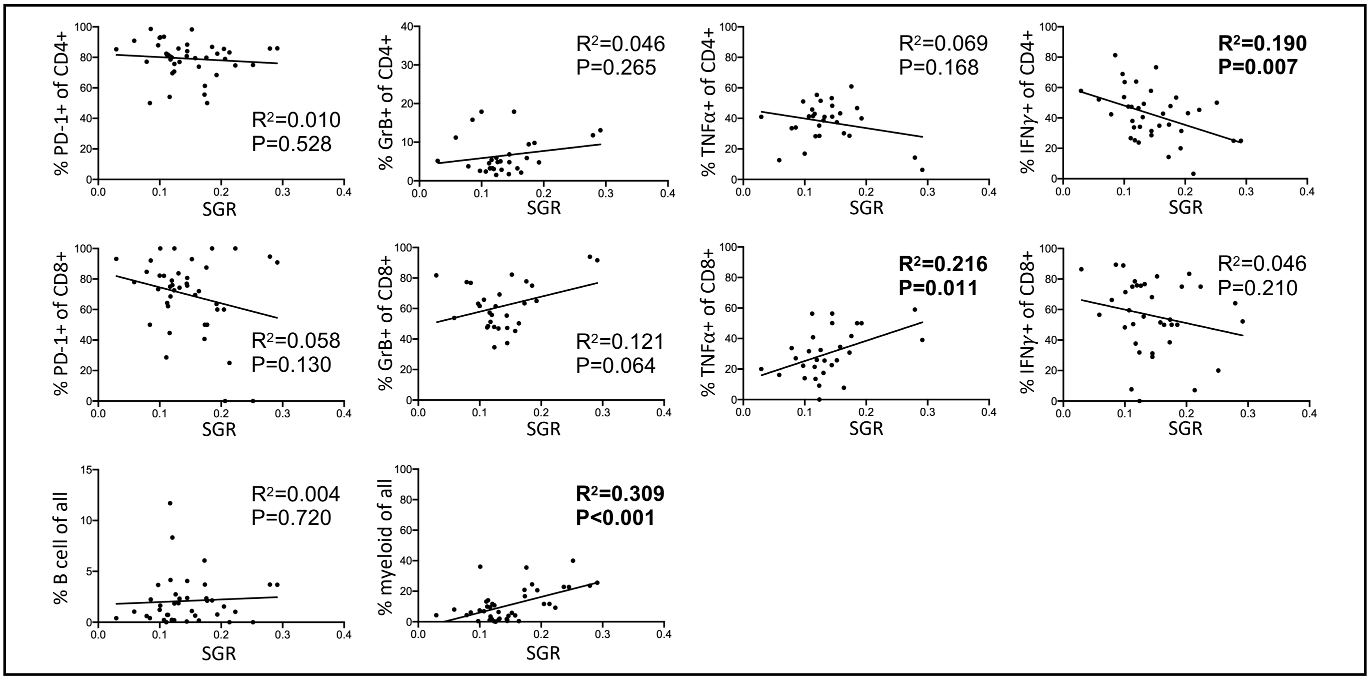

Supplementary Figure 6. Correlation of immunotypes with tumor growth (SGR, %/day) in the tumors of HIS-BRGS bearing CRC MSS PDX (B2, D2, D3, E, F) and receiving no treatment (vehicle). Immune parameter indicated on y-axis. [Linear regression analysis, R-squared score (R^2) and P value (P) in bold if statistically significant (P<0.05)].
